# Specific Medicinal Plant Polysaccharides Effectively Enhance the Potency of a DC-Based Vaccine against Mouse Mammary Tumor Metastasis

**DOI:** 10.1371/journal.pone.0122374

**Published:** 2015-03-31

**Authors:** Wei Ting Chang, Tzung Hsien Lai, Yau Jan Chyan, Shu Yi Yin, Yung Hsiang Chen, Wen Chi Wei, Ning-Sun Yang

**Affiliations:** 1 Department of Food and Nutrition, Providence University, Taichung, Taiwan, R. O. C; 2 Institute of Agricultural Biotechnology Research Center, Academia Sinica, Taipei, Taiwan, R. O. C; 3 Development Center for Biotechnology, New Taipei City, Taiwan, R. O. C; Federal University of São Paulo, BRAZIL

## Abstract

Dendritic cell (DC) vaccines are a newly emerging immunotherapeutic approach for the treatment and prevention of cancer, but major challenges still remain particularly with respect to clinical efficacy. Engineering and optimization of adjuvant formulations for DC-based vaccines is one strategy through which more efficacious treatments may be obtained. In this study, we developed a new *ex vivo* approach for DC vaccine preparation. We evaluated two highly purified mixed polysaccharide fractions from the root of *Astragalus membranaceus* and *Codonopsis pilosulae*, named Am and Cp, for their use in enhancing the efficiency of a DC-based cancer vaccine against metastasis of 4T1 mammary carcinoma in mice. Mixed lymphocyte reaction showed all Am-, Cp- and [Am+Cp]-treated DCs enhanced mouse CD4^+^ and CD8^+^ T-cell proliferation. [Am+Cp]-treated DCs exhibited the strongest anti-4T1 metastasis activity in test mice. Treatments with Am, Cp and [Am+Cp] also resulted in augmented expression of CD40, CD80 and CD86 markers in test DCs. Bioinformatics analysis of the cytokine array data from treated DCs identified that [Am+Cp] is efficacious in activation of specific immune functions via mediating the expression of cytokines/chemokines involved in the recruitment and differentiation of defined immune cells. Biochemical analysis revealed that Am and Cp are composed mainly of polysaccharides containing a high level (70–95%) glucose residues, but few or no (< 1%) mannose residues. In summary, our findings suggest that the specific plant polysaccharides Am and Cp extracted from traditional Chinese medicines can be effectively used instead of bacterial LPS as a potent adjuvant in the formulation of a DC-based vaccine for cancer immunotherapies.

## Introduction

Breast cancer is one of the most common cancers in women worldwide, and 5% to 10% of breast cancer patients are diagnosed as metastatic [1]. Each year an estimated 500,000 deaths are caused by metastatic breast cancers [2]. Metastatic breast cancers are often highly resistant to chemotherapy, and there is still little or no effective cure for patients with the advanced stage disease. Therefore, novel approaches to halt metastatic breast cancers or prolong the life of metastatic breast cancer patients are urgently needed.

Dendritic cell (DC) vaccines are a promising, newly emerging form of immune therapy for cancer. DCs are professional antigen presenting cells that have the capacity to efficiently process and present antigens, secrete immunomodulatory cytokines/chemokines, and elicit T cell responses that can be directed to generate cellular and humoral antigen-specific immunities against specific or targeted tumors, or their metastases [3]. One DC-based cancer vaccine, Provenge, was approved by the US Food and Drug Administration (FDA) in 2010 for treatment of advanced prostate cancer [4]. In addition, a novel DC-based immunotherapy directed against breast cancer-specific antigen HER2, Neuvenge, is currently being tested in early clinical trials [5]. Although some early clinical trials indicated that DC-based vaccines can increase the innate or non-specific immune responses in cancer patients, aggressive tumor progression and immune dysfunction or deficiency often limit the efficacy of such vaccines [6,7]. To overcome or reduce these difficulties, use of tumor-associated antigens or patient’s own tumor lysate as antigens is recognized as a promising approach [8]. Our recent study showed that the cell lysate derived from shikonin-treated tumor cells and LPS in combination resulted in specific DC activation, and the DC-based vaccine effectively enhanced the priming of Th1/Th17 effector cells for induction of a potent anti-tumor effect [9]. Another important issue for optimization of anti-tumor response for successful therapeutic vaccination is the active state of DCs to be delivered as cell-based vaccines [6,10]. Some studies have shown that treatment with tumor cell lysate (TCL) can inhibit the toll-like receptor (TLR)-induced maturation and function of DCs [11,12]. Mature DCs are known as potent activators of T cells, whereas immature DCs instead induce immune tolerance [13]. Effective and optimized use of an adjuvant for enhancing the potency of antigen-specific immune response is considered to be a key strategy for upgrading/enhancing the limited effect of TCL on DCs [4]. In 2009, the FDA approved the HPV vaccine, Cervarix, which is formulated with TLR agonist, monophosphoryl lipid A (MPL) as an adjuvant. The adjuvant can enhance the vaccine potency by increasing the breadth of antibody responses to HPV serotypes not contained in the vaccine [4]. MPL is the first and only TLR ligand used in a human vaccine licensed by the FDA [14]. Following the recent FDA approvals of these pioneering cell-based vaccines and cancer vaccine adjuvants, there is a need to better understand the science behind the use of TLR agonists (including specific polysaccharides) as adjuvants to aid the development of further therapeutic TCL-loaded DC-based cancer vaccines.

Although LPS is a common and highly potent TLR agonist, LPS is not permitted for clinical use due to concerns about its high cytotoxicity and systemic inflammation [15]. Our recent study demonstrated that application of a specific *Dioscorea* plant extract fraction (termed DsII), which contains mannan-rich polysaccharides, can be employed as an effective adjuvant for TCL-loaded, DC-based vaccines [16]. The DsII polysaccharides increased the maturation status of DCs, augmented the TCL-loaded DC-mediated activation of T-cell proliferation, and conferred strong anti-melanoma activity in an animal study [16].

Radix Astragalus (the root of *Astragalus membranaceus*) and Radix Codonopsis (the root of *Codonopsis pilosulae*) are well-known, commonly used, traditional Chinese medicine preparations. According to anecdotal evidence passed down for over 2000 years by practitioners of the Traditional Chinese Medicine system, both Radix Astragalus and Radix Codonopsis are most effective if taken orally, alone or in multiple-herb formulations, or as a food supplement, and promote health and homeostasis [17]. Recently, experimental and evidence-based studies have indicated that phytocompounds from Radix Astragalus may modulate immune functions. In particular, studies on enriched or highly purified *Astragalus* polysaccharides activated mouse B cells and macrophages [18], restored depressed mitogen response and inhibited leukemia and lymphoma tumor cell growth in tumor-bearing mice [19]. In addition, a number of studies have also demonstrated that the *Codonopsis* polysaccharides can suppress Treg cells and cause a shift in T-cell polarization from Th2 to Th1 responses [20]. These polysaccharides can also inhibit hepatoma growth in tumor-bearing mice and stimulate lymphocyte proliferation [21]. Moreover, accumulating evidence suggests that a formulation named Shenqi Fuzheng, a recently developed injection drug composed of phytoextracts of Radix Astragalus and Radix Codonopsis, can improve cancer treatment in advanced non-small cell lung cancer patients [22]. A study also suggested that these polysaccharides may confer bioactivities for repair or restoration of immunosuppressive activities in test mice and treated patients [23].

Radix Astragalus and Radix Codonopsis are commonly used as medicinal plants in Asia, and the mixtures of their root extracts have been reported to confer multiple and specific immune-modifier activities. In this study, we evaluated the potential application of plant polysaccharide preparations from *Astragalus membranaceus* and *Codonopsis pilosulae*, designated as Am and Cp, for *in vitro* activation of DCs in a vaccine preparation, to replace lipopolysaccharides (LPS) which are not suitable for human clinical use. Am and Cp were tested as adjuvants either alone or in combination in the formulation of a DC-based vaccine against metastasis of 4T1 mammary carcinoma in a mouse tumor-resection model. The specific cellular and molecular mechanisms likely to be involved in the observed adjuvant effect of these two polysaccharides were also investigated.

## Materials and Methods

### Mice

Female BALB/c mice aged 6–8 weeks were purchased from the National Laboratory Animal Breeding and Research Center, Taipei, Taiwan. All mice were maintained in a laminar airflow cabinet kept at 24 ± 2°C and 40–70% humidity with 12-h light/12-h dark cycles under specific pathogen-free conditions. All experimental procedures involving animals were approved by the Academia Sinica Institutional Animal Care and Utilization Committee.

### Cell lines

Mouse mammary carcinoma 4T1 and 4T1-luc2 (i.e., 4T1 cells transfected with a luciferase cDNA gene) cell lines were kindly provided by Dr. Pei-Wen Hsiao (Academia Sinica, Taipei, Taiwan) [24]. Both cell types were maintained in RPMI-1640 complete medium (i.e., RPMI-1640 supplemented with 10% FBS, 100 μM non-essential amino acids, 100 μM sodium pyruvate, 100 μg/ml streptomycine and 100 unit/ml penicillin) and grown in a 5% CO_2_ incubator at 37°C before use. The stable clones of transfected 4T1-luc2 cells were selected in medium further supplemented with 0.5% puromycin.

### Generation of bone marrow-derived dendritic cells

Mouse bone marrow-derived DCs (BMDCs) were generated and modified as previously described [25]. Briefly, bone marrow tissues were collected from the femurs and tibiae of BALB/c mice. Erythrocytes were removed from the derived bone marrow cells by ammonium-chloride-potassium (ACK) lysis buffer and plated in 30 ml RPMI 1640 complete medium supplemented with 20 ng/ml GM-CSF at 37°C in a 5% CO_2_ atmosphere in an incubator. On day 2, two-thirds of the original medium was removed and 30 ml fresh complete medium containing the same amount of GM-CSF was added. On day 5, the floating cells were gently removed and 30 ml fresh complete medium containing 20 ng/ml GM-CSF and 20 ng/ml IL-4 was used to replenish the culture. On day 7, the floating and loosely adherent DCs in the culture were harvested for subsequent analyses. The purity of these DC populations was determined by flow cytometry analysis. Our culture conditions routinely resulted in the production of ≥ 85% MHC-II^+^ and CD11c^+^ DCs from bone marrow cells of 5- to 6-week old mice (S1A Fig).

### Preparation of tumor cell lysate

Mouse mammary carcinoma 4T1 cells were collected and resuspended in PBS at a density of 5 × 10^7^ cells/ml. Test cells in suspension were frozen in liquid nitrogen and subjected to four freeze-thaw cycles [8]. After the final cycle, the cell lysate suspensions were centrifuged at 13,000 rpm for 20 min and the supernatants were used as the source of tumor antigen.

### Preparation of Cp, Am and [Am+Cp] phytoextracts

The plant tissues of Radix Astragalus (root of *Astragalus membranaceus* (Fisch.) Bunge var. *mongholicus* (Bunge) P. K. Hsiao) and Radix Codonopsis (root of *Codonopsis pilosulae*) were extracted with ddH_2_O (1:10; weight:volume) at 85°C for 2 h, and the supernatant of the extract preparation was successively filtered with 0.2 μm hollow-fiber filter and a 10 kDa ultrafiltration membrane. Subsequently, the enriched >10 kDa fraction was concentrated by a falling film concentrator and further extracted with 95% ethanol at the fraction/ethanol ratio of 1:4 at room temperature for 16 h. All materials in the resultant ethanol-partitioned fraction were determined to have a molecular mass of more than 10 kDa by gel permeation chromatography using a Shodex KS-804 column (data not shown). The resultant ethanol-partitioned fraction was lyophilized for use as our experimental material. The two phytoextracts of Radix Astragalus and Radix Codonopsis were designated as Am and Cp, respectively. A mixture of the Am and Cp sample preparations, at a ratio of 1:1, termed [Am+Cp], was also used for this study.

### Cell viability assay

To evaluate the possible cytotoxic effect of candidate adjuvants and TCL, 3-(4,5-Dimethythiazol-2-yl)-2,5-diphenyltetrazolium bromide (MTT) colorimetric assay was performed on treated cells. Briefly, BMDCs were seeded into 96-well plates at a density of 2 × 10^5^/200 μl/well and incubated with candidate adjuvants (i.e., Cp, Am or [Am+Cp]) at doses between 1 and 1000 μg/ml or TCL at concentrations between 50 and 500 μg/ml for 24 h in RPMI 1640 complete medium. At the end of the incubation period, the culture plates were centrifuged for 5 min at 1200 rpm for pelleting of DCs. And then the test culture media were removed and the DCs were incubated with 100 μl MTT solution (0.5 mg/ml) for 4 h at 37°C. The MTT reduction by living cells was confined to the intracellular formazan granules and the needle-like formazan crystals localized on the cell surface were generated as exocytosed MTT formazan (S2 Fig). Subsequently, the MTT solution was removed and the blue formazan crystals trapped in DCs were dissolved in 200 μl DMSO and incubated for 30 min. Absorbance at 570 nm was measured by a multiwell scanning spectrophotometer. Experiments were performed in triplicate samples. The percentage of viability was calculated using the formula: “Mean OD (optical density) of treated cells/mean OD of untreated cells (control) × 100”.

### Activation of TCL-loaded DCs with Cp, Am or [Am+Cp] phytoextracts

After cultivation for 7 days (see above), DCs were collected and washed three times with complete RPMI 1640 medium. Subsequently, DCs (5 × 10^6^ cells/6 ml) were incubated for 2 h with TCL containing 200 μg protein/ml. Aliquots (60 or/and 120 μl) of Am, Cp or [Am+Cp] (10 mg/ml in stock) were then added to make a final concentration of 100 or/and 200 μg/ml of test compound(s) in 6 ml culture medium, and cell cultures were incubated for another 22 h. Treatment with 1 μg/ml LPS was used as a positive control. After treatment with Cp, Am, [Am+Cp] or LPS, test DCs were used to evaluate the expression of maturation markers (i.e., CD40, CD80 and CD86) (BD Pharmingen, San Diego, CA, USA) using flow cytometry analyses, stimulatory activity of T-cell proliferation by mixed lymphocyte reaction (MLR) assay, and the anti-metastasis effect of 4T1 mammary tumor was evaluated by *in vivo* mouse study.

### Mixed lymphocyte reaction

The splenocytes and splenic T-cells (CD4^+^ and CD8^+^ T-cells) to be used as responders were isolated from spleens of BALB/c mice. For CD4^+^ and CD8^+^ T-cell purification, a magnetic-activated cell sorting (MACS) selection of splenocytes with CD4 or CD8 antibody-conjugated microbeads (Miltenyi Biotec; resulting in >98% purity and >98% viability) were performed. Co-cultivation of the DC-splenocyte or-T cell system was performed as previously described [26]. Briefly, a total of 1 × 10^5^ splenocytes, CD4^+^ or CD8^+^ T cells were co-cultured with BMDCs for 4 days in a final volume of 200 μl medium at a DC/splenocyte or DC/T-cell ratio of 1:100, and then BMDC-induced splenocyte or T-cell proliferation activity was determined by Cell Proliferation ELISA (Roche Applied Science, Indianapolis, IN).

### Flow cytometry analysis of cell surface markers

After test DCs were incubated with TCL and candidate adjuvants or LPS-treated (see above) DC samples were washed and re-suspended in wash buffer (i.e., PBS supplemented with 1% BSA and 2 mM EDTA) and seeded into a 96-well plate (10^5^ cells/well). For blocking of Fc receptors, DCs were pre-incubated with purified anti-CD16/CD32 antibody (Pharmingen, San Diego, CA) for 15 min at 4°C. Subsequently, test cells were stained with fluoresceinisothiocyanate (FITC)-conjugated anti-mouse CD40, CD80, CD86 and MHC class II, or with phycoerythrin (PE)-conjugated anti-mouse CD11c and MHC-II antibodies (BD Pharmingen, San Diego, CA, USA) for another 30 min at 4°C. Cells were washed twice with wash buffer and resuspended in a 4% paraformaldehyde solution. The phenotypic change of test DCs was analyzed with a BD LSR II flow cytometer and BD FACSDiva software (BD, Franklin Lakes, NJ). The four parameters simultaneously collected were forward scatter (FSC), side scatter (SSC), log FITC and log PE. The gating scope (P9) was gated to exclude debris and aggregated cells on a FSC/SSC histogram (S1B Fig); 10,000 cells were counted in P9 and data were recorded.

### Cytokine array

BMDCs (5 × 10^6^/6 ml) were incubated with 200 μg protein/ml TCL for 2 h and then treated with Cp, Am, [Am+Cp] (200 μg/ml) or LPS (1 μg/ml) (positive control) for another 22 h. Conditioned culture media of DCs were collected and analyzed by Mouse Cytokine Array Panel A (R&D Systems, Minneapolis, MN). A total of 40 cytokines/chemokines were analyzed in this assay, their names and cytokine array panel coordinates are provided in S3 Fig. The assays were performed according to the manufacturer’s protocol. Briefly, aliquots of 15 μl cytokine array detection antibody cocktail were added to each test sample and incubated at room temperature for one hour. Cytokine array membranes were then incubated with sample/antibody mixtures overnight at 4°C on a shaker. Test samples on membranes were washed 3 times with 1X wash buffer, each for 10 minutes. Membranes were then incubated with Streptavidin-HRP solution for 30 minutes at room temperature on a shaker. Membranes were washed 3 times again as before. Aliquots of 1 ml Chemi Reagent Mix were added onto each membrane and incubated for 1 minute. The membrane was drained and exposed to X-ray film for 1–10 minutes. Data were analyzed using Alpha View SA 3.4 software. Mean values of spotted duplicates were calculated and normalized with the mean values of reference spots (i.e., internal positive controls). Cytokines/chemokine expression levels were indicated as fold-change when compared to those of the control group (DC+TCL).

### Vaccine sample preparation

After cultivation for 7 days (see “Generation of bone marrow-derived dendritic cells” section), DCs were harvested and washed three times with complete RPMI 1640 medium. DC samples were then seeded in a 15-cm dish at 1.5 × 10^7^ cells/dish and treated with TCL at 200 μg/ml for 2 h. The TCL-loaded DCs were subsequently activated by candidate adjuvants (i.e., Cp, Am or [Am+Cp]) at 200 μg/ml or LPS (positive control, 1 μg/ml) for another 22 h. At the end of this incubation, activated DCs were collected and washed three times with PBS and adjusted to a cell density of 5 × 10^6^ cells/ml PBS, ready for use in mouse vaccination.

### 4T1 mammary carcinoma model

Mice were subcutaneously injected with 4T1-Luc2 cells (5 × 10^5^/50 μl PBS/mouse) into the fourth mammary fat pad under 2.5% isoflurane anesthesia. Tumor growth was monitored by measuring the tumor volume according to the formula: volume = length × (width)^2^/2. After tumors were established (180–200 mm^3^) on day 15, the mice were divided into seven groups (8 mice/group) and subjected to different treatment regimens: (1) PBS (untreated control), (2) DC, (3) DC+TCL, (4) DC+TCL+Cp, (5) DC+TCL+Am, (6) DC+TCL+[Am+Cp] and (7) DC+TCL+LPS (positive control). On day 16 after tumor cell implantation, the primary tumors were surgically resected and incisions were closed with sutures under 2.5% isoflurane anesthesia. The seven groups of mice were then vaccinated by priming and boosting with test vaccines (1 × 10^6^ DCs/200 μl PBS/mouse) via intravenous injection at 1, 8 and 15 days post tumor resection. To monitor the progression of metastatic cancers, bioluminescence signals of the 4T1-luc2 tumor cells in test mice were imaged by using a non-invasive *in vivo* imaging system (IVIS) (Xenogen Corp, Alameda, CA, USA) after the intraperitoneal injection of 150 mg/kg D-luciferin (NanoLight technology, Pinetop, AZ, USA). When mice were unable to ambulate to reach food/water, or displayed signs of respiratory distress due to tumor metastasis, the mice were sacrificed (CO_2_ inhalation) and the date was recorded to calculate survival rate. The criteria used to determine humane endpoint were strictly based on the Guidelines for Determining Endpoints and Humane Termination of Animals provided by the Institutional Animal Care and Use Committee (IACUC) of Academia Sinica, Taiwan (S1 File). All test mice were euthanatized by CO_2_ inhalation at the end of the experiment.

### Hematoxylin and eosin (H&E), and immunohistochemistry staining (IHC) staining

Lung tissues were collected from test mice and fixed with 10% formalin, embedded in paraffin. After tissue samples were deparaffinized and rehydrated, tissue samples were subjected to hematoxylin and eosin (H&E) and immunohistochemistry staining (IHC). For immunohistochemistry staining, antigen retrieval was performed with 0.01 M citrate buffer at pH 6.0, and heated for 20 min. Cooled tissue slides were rinsed three times in PBST buffer (0.05% Tween 20 in PBS buffer). Endogenous peroxidase activity was blocked using PBST buffer containing 3% hydrogen peroxide for 5 min. Tissue sections were then incubated with a primary rat anti-mouse CD8a antibody (BD, San Diego, CA, USA) overnight at 4°C, and subsequently with secondary HRP-conjugated anti-rat IgG antibody (Santa Cruz, Dallas, Texas, USA). Immunoreactivity was visualized by the diaminobenzidine (DAB) substrate kit (BD, San Diego, CA, USA). All test lung tissue sections were counterstained with hematoxylin.

### Functional pathway analysis

Ingenuity Pathway Analysis (IPA) software (www.ingenuity.com) was used to analyze the functional pathways/networks of the Cp-, Am-, [Am+Cp]- and LPS-induced, differentially expressed cytokines/chemokines in test DCs that were identified by the cytokine array analysis. Only cytokines and chemokines with expression levels altered more than two-fold in both replicate arrays were screened and selected by IPA for functional pathways analysis.

### Monosaccharide composition analysis

Analysis of the monosaccharide (sugar) composition in Radix Astragalus and Radix Codonopsis polysaccharides (i.e., Am and Cp) was performed as previously described [27] with slight modification. Briefly, monosaccharides were separated by a high-performance anion-exchange chromatography (HPAEC) system (DX 500 Chromatography System, Dionex, USA), equipped with a pulsed amperometric detection (PAD) (ED40, Dionex, USA) device and a gradient pump (GP40, Dionex, USA). For sample preparation, 0.5 mg/ml of Cp or Am sample was dissolved in ddH_2_O containing 0.2 mM allose (used as an internal control), filtered through a PVDF filter (0.22 μm) and hydrolyzed with 2N trifluoroacetic acid. The 20 μl of Cp or Am hydrolysate was injected into a CarboPac PA-1 guard column (4 mm × 50 mm, Dionex, USA) attached to an anion-exchange column (4 mm × 250 mm, Dionex, USA). For gradient elution, buffer A [8.75 mM Ba(OH)_2_] and buffer B [100 mM NaOH, 100 mM NaOAc, 2 mM Ba(OH)_2_] solutions were used as the mobile phase at a flow rate of 1 ml/minute. Data were collected and analyzed by a computer equipped with Dionex PeakNet software. D-Glucose monohydrate (Glu), D-Glucuronic acid (GluA), D-(+)-Galactose (Gal), D-(+)-Allose (All), D-(-)-Arabinose (Ara), L-(+)-Rhamnose monohydrate (Rhm), and D-(+)-Galacturonic acid (GalA) were hydrolyzed in the same way as mentioned above and used as the standard/reference monosaccharides.

### Statistical analysis

Data were analyzed by one-way or two-way ANOVA. Statistical analyses were conducted with GraphPad Prism 5.0 (San Diego, CA). Differences in mouse survival time and rate were determined by a log-rank (Mantel-Cox) test of the Kaplan-Meier survival curves. All statistical tests were two-sided. A *P* value of less than 0.05 was considered significant (*, *P* < 0.05; **, *P* < 0.01; ***, *P* < 0.001; n.s, no significance).

### Ethics statement

All animal studies for this research work were performed in strict accordance with the recommendations in the Guide for the Institutional Animal Care and Use Committee (IACUC) of Academia Sinica. The protocol was approved by the IACUC of Academia Sinica (Protocol ID: 12-01-304) (S2 File). This research complies with the Animal Research: Reporting of In Vivo Experiments (ARRIVE) guidelines (S1 ARRIVE Checklist).

## Results

### TCL-loaded DCs with polysaccharide mixtures Cp, Am and [Am+Cp] drastically enhance proliferation of T-cells

**Fig 1:**
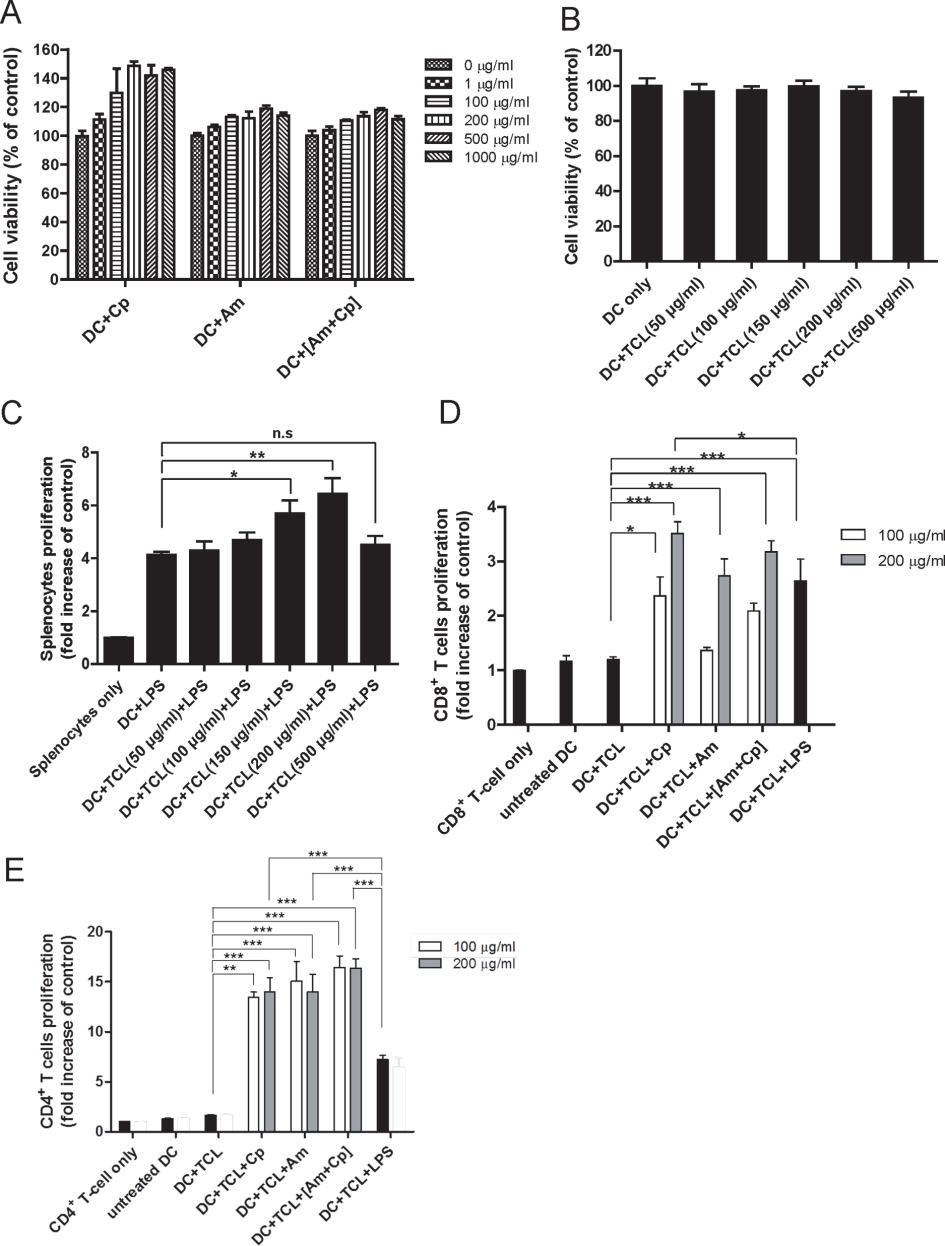
Effect of candidate adjuvants and tumor cell lysate on cell viability and T-cell proliferation. (A) Effect of candidate adjuvants (i.e., Cp, Am and [Am+Cp]), and (B) tumor cell lysate on viability of dendritic cells. DCs (2 × 10^5^) were treated with Cp, Am or [Am+Cp] at a dose between 1 to 1000 μg/ml or treated with TCL at concentrations between 50 and 500 μg/ml for 24 h. Cell viability was performed by MTT assay. Data represent the mean ± SD of three replicates. Optimal dosage/concentration of TCL, Cp, Am and [Am+Cp] phytoextracts for stimulating DC-mediated activation of splenocyte or T-cell proliferation. (C) DCs as stimulator cells were pulsed with TCLs at 50–500 μg/ml in medium supplemented with LPS at 1 μg/ml. (D) and (E), TCL-loaded DCs as stimulator cells were treated with Cp, Am or [Am+Cp] phytoextracts at 100 or 200 μg/ml, or with 1 μg/ml LPS (positive control). The (C), (D) and (E) sets of DC stimulator cells were then co-cultured with splenocytes (C), CD8^+^ T cells (D) or CD4^+^ T cells (E), as responder cells, for 4 days. Cell proliferation activities of (C) splenocytes, (D) CD8^+^ cell and (E) CD4^+^ cells are represented as the fold change over control (i.e., splenocytes or T cells only). Data represent the mean ± SE obtained from three independent experiments. A *P* value of less than 0.05 was considered significant (*, *P* < 0.05; **, *P* < 0.01; ***, *P* < 0.001; n.s, no significance).

In order to characterize the applicability, and biosafety of the candidate adjuvants for a DC-based cancer vaccine, the cytotoxicity of Cp, Am and [Am+Cp] and 4T1 tumor cell lysate on test DCs was determined by MTT assay. Cp, Am and [Am+Cp] at a dose between 1 and 1000 μg/ml did not impair the viability of test DCs after treatment for 24 h (Fig 1A). In addition, TCL at a concentration between 50 and 500 μg/ml also did not exhibit a cytotoxic effect on test DCs (Fig 1B).

We previously showed that the anti-melanoma efficiency of a DC-based cancer vaccine is mainly by activation of T-cell expansion and activation [16]. Thus, T-cell proliferation activity was used in this study to evaluate and optimize the efficiency of the candidate adjuvants (i.e., Cp, Am and [Am+Cp]) and TCL on DC activation. When compared with the control group (DC+LPS), TCL at concentrations of 150 and 200 μg/ml resulted in a significant increase in splenocyte proliferation (Fig 1C). However, when dosage of test TCLs was increased to 500 μg/ml, the level of splenocyte proliferation was significantly decreased. We further evaluated the cytotoxic effect of TCL on splenocytes. We observed that at a dose of TCL at 500 or 1000 μg/ml, cell viability of treated splenocytes was indeed slightly suppressed, down about 10% to 15% (S4 Fig). We, therefore, consider that it is logical to think that there may indeed be other factors that can contribute to the low level of splenocyte proliferation activity at this relatively high concentration (≥ 500 μg/ml) of TCL treatment. Presumably this could be due to an inhibitory effect of the high concentration of TCL (≥ 500 μg/ml) used on DCs, affecting maturation and other cellular activities of DCs [11,12].

A previous study on DC-based cancer immunotherapy focused on the activation and expansion of tumoricidal CD8^+^ cytotoxic T cells [10]. Since CD4^+^ T cells are also known to play a role in orchestrating antibody production and the activation and expansion of CD8^+^ T cells [28], and increasing evidence has shown that CD4^+^ T cells can significantly contribute to tumor suppression *in vivo* [29,30], in the present investigation, we evaluated whether CD8^+^ and CD4^+^ T-cell proliferation activities could also be affected by vaccination with Cp, Am or [Am+Cp]-treated, TCL-loaded DCs. As seen in Fig 1D, both Cp and [Am+Cp] extracts, at 100 and 200 μg/ml, significantly enhanced CD8^+^ T-cell proliferation activity, as compared with that of the DC+TCL set. Treatment with Am only at 200 μg/ml effectively increased CD8^+^ T-cell proliferation activity (Fig 1D). As a dose response, there was a 1.5- to 2.0-fold difference in activity between 100 μg/ml and 200 μg/ml for all three (Cp, Am and [Am+Cp]) treatment groups (Fig 1D). In addition, the Cp, Am and [Am+Cp] phytoextracts at concentrations of 200 μg/ml induced 2.9-, 2.3- and 2.6-fold higher CD8^+^ T-cell proliferation activity than the control group (DC+TCL). When compared to DC+TCL+LPS, only 200 μg/ml of DC+TCL+Cp significantly increased the CD8^+^ T-cell proliferation. In comparison, neither the Am, the [Am+Cp] group (at doses of 100 and 200 μg/ml) nor the Cp group (at 100 μg/ml) showed a statistically significant difference in proliferation level activities of CD8^+^ T cells in comparison with the LPS group (Fig 1D). Furthermore, as shown in Fig 1E, these three phytoextracts (i.e., Cp, Am, and [Am+Cp]) at doses of 100 μg/ml and 200 μg/ml induced similarly high levels of DC-mediated CD4^+^ T-cell proliferation, i.e., 8.2- to 10.0-fold higher levels than that of the control group (DC+TCL). Taken together, these three phytoextracts at 200 μg/ml showed a consistently high level of induction of CD8^+^ and CD4^+^ T-cell proliferation. The 200 μg/ml dose of test phytoextracts was, therefore, used as the optimal dose in subsequent *in vitro* and *in vivo* studies.

### [Am+Cp] polysaccharide combination as an adjuvant suppresses mammary tumor metastasis and improves mouse survival in an orthotopic tumor resection model

**Fig 2:**
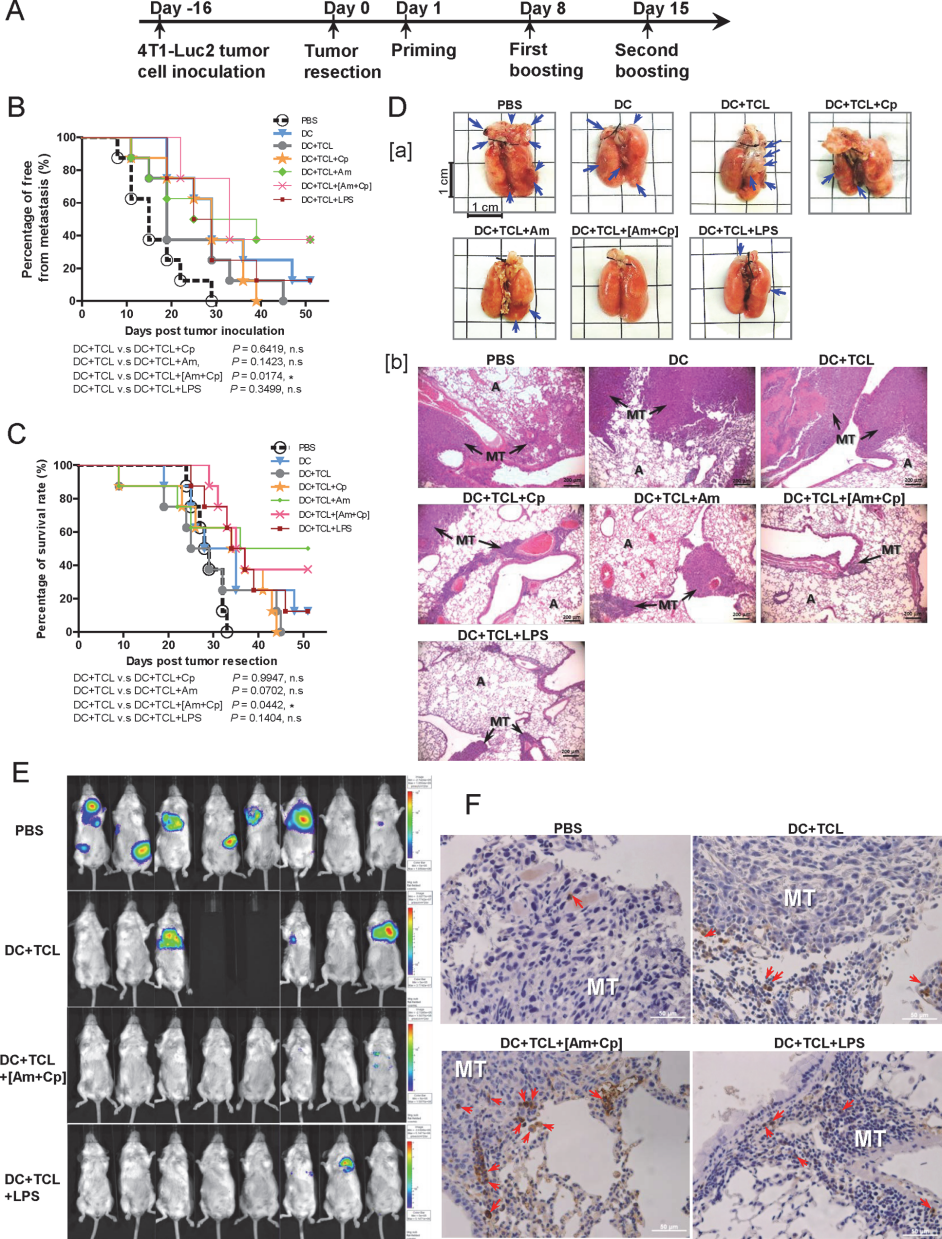
Adjuvant effect of [Am+Cp] phytoextract on DC vaccine against *in vivo* metastasis of 4T1 mammary tumors. (A) Treatment schema used for the cancer vaccine experiment. (B) Percentage of whole body organs free from metastasis. (C) Survival rate of treated 4T1 tumor-bearing mice after resection of primary tumor(s). The free from tumor metastasis rate and mouse survival rate were statistically analyzed at day 51 after resection of the primary tumor. (D[a]) Metastatic pulmonary foci of 4T1 (shown as arrows) in lung were detected in 4T1 tumor-resected mice. (D[b]) Histological staining of tumor-bearing lung tissue sections with H&E. Arrows indicate metastatic tumors (MT: metastatic tumors; A: alveolus). (E) Bioluminescence imaging of the whole mouse body obtained with an in vivo imaging system (IVIS). (F) Immunohistochemistry staining for CD8^+^ T cells in lung tissue section. Arrows indicate the infiltrating CD8^+^ T cells in the tumor site of the lung tissues (MT: metastatic tumors).

Based on the results shown in Fig 1, we hypothesized that the anti-tumor effect of a DC vaccine may be enhanced by treatment with Cp, Am and [Am+Cp] plant polysaccharides, in a similar manner to treatment with LPS. We, therefore, tested Cp, Am and [Am+Cp] as potential activators/adjuvants of a DC-based vaccine formulation to inhibit tumor metastasis and regional tumor growth in a mouse 4T1 mammary tumor-resection model. At 1, 8 and 15 days post tumor resection, test mice were tail vein-injected with different phytoextract-stimulated vaccine regimes (see experimental design schema, Fig 2A). The DC+TCL+[Am+Cp] group showed the most significant inhibition of tumor metastasis, as compared to the DC+TCL group (Fig 2B). Furthermore, 50% of mice treated with DC+TCL vaccine had died due to metastasis on day 28 (Fig 2C). In contrast, all (100%) of the DC+TCL+[Am+Cp] vaccine-treated mice were still alive on day 28 (Fig 2C). The day 25 to day 29 time window was observed to have particularly high levels of metastatic activity, and hence it is important to address the effect of DC+TCL+[Am+Cp] versus other treatment groups over this time period. Indeed, DC+TCL+[Am+Cp] vaccine exhibited the most efficacious anti-metastasis activity (*P* < 0.044) and prolonged the survival time of test mice (*P* < 0.0273) during this day 25 to day 29 time period of observation (Fig 2B and 2C). This differentiation phase may be a critical period for complementary treatment. The DC+TCL+[Am+Cp] treatment group showed a drastic decrease in the number of metastatic pulmonary foci at the gross anatomy level, when compared with the DC+TCL group, as observed on day 22 (Fig 2D[a]). At the tissue level, histological analysis showed that lung tissues from the DC+TCL+[Am+Cp] group exhibited smaller and fewer metastatic tumor foci when compared to the control group (DC+TCL) (Fig 2D[b]). The Cp, Am and LPS treatment groups, in comparison to DC+TCL+[Am+Cp] group, showed relatively less reduction in metastasis. Fig 2E shows representative images of 4T1-luc2 tumor-bearing mice from different test groups at day 22 post tumor resection. The bioluminescence intensities are indicative of tumor volumes. The whole-body bioluminescent images of the PBS and DC+TCL-treated groups show that after tumor resection, tumor cells metastasized and grew mainly into the lung region, although they also disseminated into other organs (e.g., liver, spleen and left-side of fat pad) (Fig 2E). One mouse from the PBS group showed a tumor recurrence or relapse at the primary tumor site (the fourth mammary fat pad on the right-side) (Fig 2E). For the mouse group treated with DC+TCL+[Am+Cp] vaccines, tumor imaging analyses showed that very limited numbers tumor cells had metastasized into lung tissues, very few or none were disseminated to other organs (Fig 2E). In comparison, mice treated with DC+TCL+LPS exhibited metastasis at a slightly higher level, apparently only into lung (Fig 2E). Furthermore, [Am+Cp] treatment can apparently increase the accumulation of CD8^+^ T cells at the lung tissue tumor site (Fig 2F), and this activity may be associated with the enhanced antitumor activity observed for the DC-based vaccine. Together, these data show that the [Am+Cp], as a DC-based vaccine adjuvant, can exhibit a significant anti-metastatic effect in a 4T1 tumor resection model (*P* = 0.0174) and prolong the survival time (*P* = 0.0442) of test mice, as scored at the end of the *in vivo* experiment (i.e., day 51 post tumor resection), as compared to mice treated with DC+TCL. In terms of both anti-metastasis activity and mouse survival rate, no statistical significance was observed among the DC+TCL+Cp, DC+TCL+Am, and DC+TCL+LPS treatment groups, as compared to those of the DC+TCL group, Fig 2B and 2C, respectively.

### Cp, Am and [Am+Cp] promote maturation of TCL-loaded DCs

**Fig 3:**
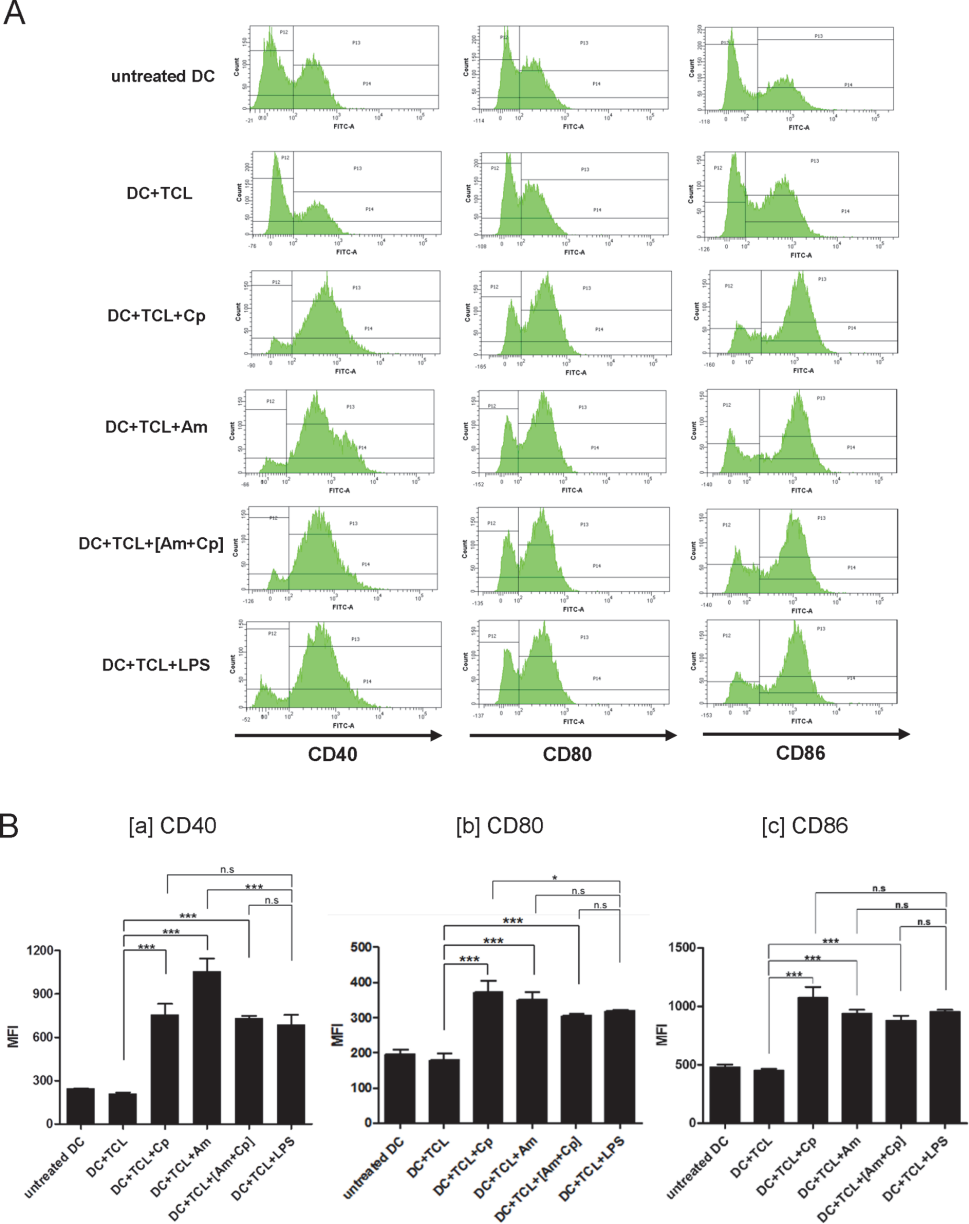
Flow cytometric analysis of expression of surface markers on DCs with different treatments. (A) Phenotypic changes in expression of CD40, CD80 and CD86 maturation markers in TCL-loaded DCs as a response to treatment with Cp, Am or [Am+Cp] phytoextracts, all at a concentration of 200 μg/ml. Anti-CD40, CD80 or CD86 antibodies were conjugated with FITC. (B) The mean fluorescence intensity (MFI) of (B[a]) CD40, (B[b]) CD80, or (B[c]) CD86 in DCs from different groups were calculated and are presented as a bar chart. Data represent the mean ± SD obtained from three independent experiments. A *P* value of less than 0.05 was considered significant (*, *P* < 0.05; **, *P* < 0.01; ***, *P* < 0.001; n.s, no significance).

Based on the findings of the *in vivo* study (Fig 2), we next evaluated the functional effect of Cp, Am and [Am+Cp] on mouse DCs. Maturation activity is an essential requirement for DCs to prime T cells and induce potent immune responses, we hence assayed the phenotypic changes of three specific molecular markers (CD40, CD80 and CD86) for maturation of DCs. DCs treated with TCL alone (i.e., DC+TCL) were not able to induce DC maturation (Fig 3). In combination with Cp, Am or [Am+Cp], the TCL-loaded DCs showed significantly increased expression of CD40, CD80 and CD86, with a 1.7- to 4.5-fold increase over that of the control group cells (DC+TCL). Among these three groups, Am treatment (DC+TCL+Am) resulted in the most significant increase in CD40 expression, whereas expressions of CD80 and CD86 markers were most upregulated in the Cp group (DC+TCL+Cp). Importantly, all the levels of stimulatory activity were similar or higher than those detected for the positive control (+LPS) group.

In addition, we also observed that this Cp, Am and [Am+Cp] can increase the population of MHC class II^high^ DCs. The result showed that DC+TCL treated with Cp, Am or [Am+Cp] increase the MHC class II^high^ population more, almost 3.6% to 6.8% over the control group (DC+TCL) (S5 Fig).

### Treatment of TCL-loaded DCs with Cp, Am and [Am+Cp] results in upregulation of expression of a spectrum of immunomodulatory cytokines and chemokines

**Fig 4:**
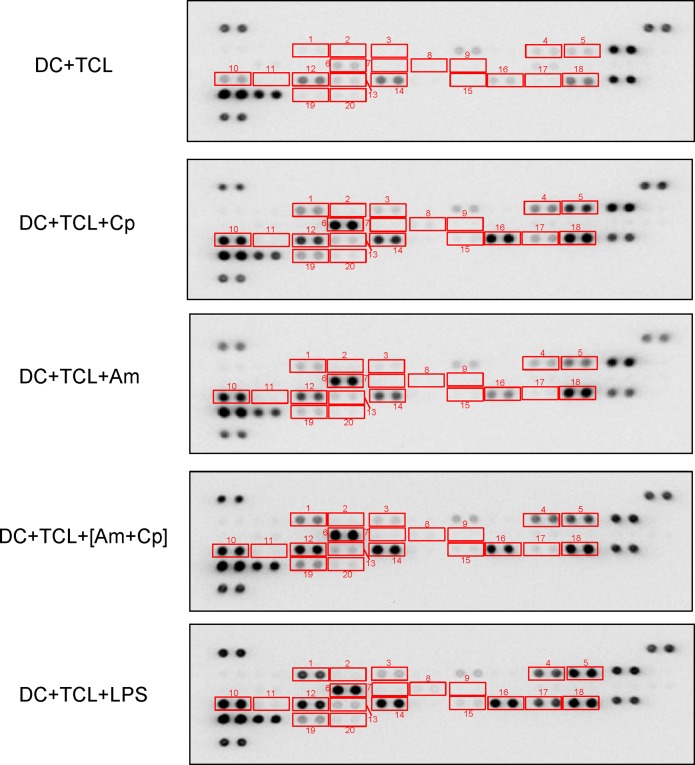
Cytokine array/profiling analysis of the alteration of multiple cytokines/chemokines in conditioned culture media of Cp-, Am-, [Am+Cp]- and LPS-treated DCs. Cytokine array membranes were incubated with cultured media from DCs that were treated with TCL for 2 h, and then with Cp, Am, [Am+Cp] or LPS for another 22 h. Squares mark the cytokines and chemokines secreted from DCs that were increased in Cp, Am, [Am+Cp] or LPS treatments: **1.** CSF3, **2.** CSF2, **3.** CCL1, **4.** IL-1α, **5.** IL-1β, **6.** IL-5, **7.** IL-6, **8.** IL-7, **9.** IL-10, **10.** CXCL10, **11.** CXCL11, **12.** CXCL1, **13.** M-CSF, **14.** CCL2, **15.** CXCL9, **16.** CCL3, **17.** CCL4, **18.** CXCL2, **19.** TNF-α, **20.** TREM-1.

Previous studies have shown that a spectrum of cytokines and chemokines effectively mediate innate and adoptive immune responses against tumor growth and metastasis through recruitment of DCs, monocytes, neutrophils, and T lymphocytes to the specific tumor sites [31,32]. TNF-α, IL-6, CCL1, CCL3, CXCL1, CXCL2, CXCL10 and others are known to play key roles in recruiting leukocytes or DCs into circulation and enhancing various cellular immune functions [32,33]. In addition, DCs can mediate T-cell differentiation and polarization through the expression and release of specific cytokines, such as IL-2 (T-cell differentiation cytokine), IL-12 (Th1-polarizing cytokine), IL-10 (Th2-polarizing cytokine), IL-23, IL-1β and IL-6 (Th17-polarizing cytokine) [9,16]. Therefore, to characterize the functional effect of Cp, Am and [Am+Cp] on TCL-loaded DCs, we analyzed the level of a series of immunomodulatory cytokines and chemokines in the conditioned media of test DCs by cytokine array analysis. Our data shows that when compared with the control group (DC+TCL), Cp-, Am- and [Am+Cp]-treated DCs induced the expression of a range of immune-relevant regulator/modifier proteins, including CSF3 (6.82- to 10.60-fold), CCL3 (4.96- to 11.15-fold), CCL1 (5.93- to 7.11-fold), TNF-α (4.32- to 6.99-fold), CXCL10 (4.8- to 6.82-fold), CXCL2 (3.62- to 5.81-fold), CCL2 (1.80- to 3.31-fold), CXCL1 (1.72- to 3.12-fold) and CCL4 (2.66- to 6.15-fold) (Fig 4 and Table 1). Most of these proteins are mainly involved in the recruitment of a variety of immune cells. Furthermore, among the test cytokines and chemokines known to be involved in T-cell differentiation and polarization, IL-1β and IL-6 were found to be upregulated, exhibiting 5.38- to 6.36-fold and 10.23- to 14.18-fold changes in expression, respectively (Fig 4 and Table 1). The trend in cytokine and chemokine expression profile change in the three test groups (i.e., Cp, Am and [Am+Cp]) was, in general, similar, and these changes were also quite similar to those of the LPS group. The fold-change in expression of the top 20 most significantly upregulated cytokines and chemokines (in [Am+Cp]-treated DCs) are presented in Table 1. The stimulation activity (in fold change) of all of the 40 cytokines/chemokines in the test DC samples is listed in S1 Table. Taken together, data from the cytokine/chemokine profiling suggest that the key functional effect of Cp, Am and [Am+Cp] on treated DCs is augmentation of the capacity of DCs to recruit immune cells and polarize T cells.

**Table 1:** Stimulation (in fold change) of cytokines and chemokines in DCs-treated with Cp, Am, [Am+Cp] or LPS groups compared to control group.

**Cytokines/Chemokines**	**Alternate Nomenclature**	**Cp**	**Am**	**[Am+Cp]**	**LPS**
IL-6	—	10.23	14.18	12.12	9.95
CSF3	G-CSF	7.37	6.82	10.60	15.58
CCL3	MIP-1α	11.15	4.96	9.16	10.74
CCL1	I-309/TAC-3	6.61	5.93	7.11	9.03
TNF-α	—	4.62	4.32	6.99	5.16
IL-1β	IL-1F2	6.36	5.89	5.38	9.86
IL-1α	IL-1F1	3.75	3.27	4.95	6.08
CXCL10	IP-10/CRG-2	4.85	6.82	4.80	5.59
CXCL2	MIP-2	3.62	5.81	3.62	3.31
CCL2	MCP-1	1.80	2.08	3.31	2.71
CXCL9	MIG	3.24	2.90	3.12	4.13
CXCL1	KC	1.72	2.37	3.12	2.38
IL-5	—	2.42	3.92	3.11	2.29
CCL4	MIP-1β	6.15	2.66	2.68	18.41
IL-7	—	1.85	3.05	2.64	1.89
CSF2	GM-CSF	2.51	4.31	2.49	2.27
CSF1	M-CSF	1.46	1.93	2.35	1.57
TREM-1	—	1.24	1.43	2.17	1.33
IL-10	—	2.63	1.96	2.02	2.59
CXCL11	I-TAC	1.76	2.96	1.90	1.93

* Fold change = treatment group/control untreated group

** Control group: DC+TCL

### Bioinformatics analysis of plant polysaccharide-induced, differentially expressed cytokines/chemokines in DCs

**Fig 5:**
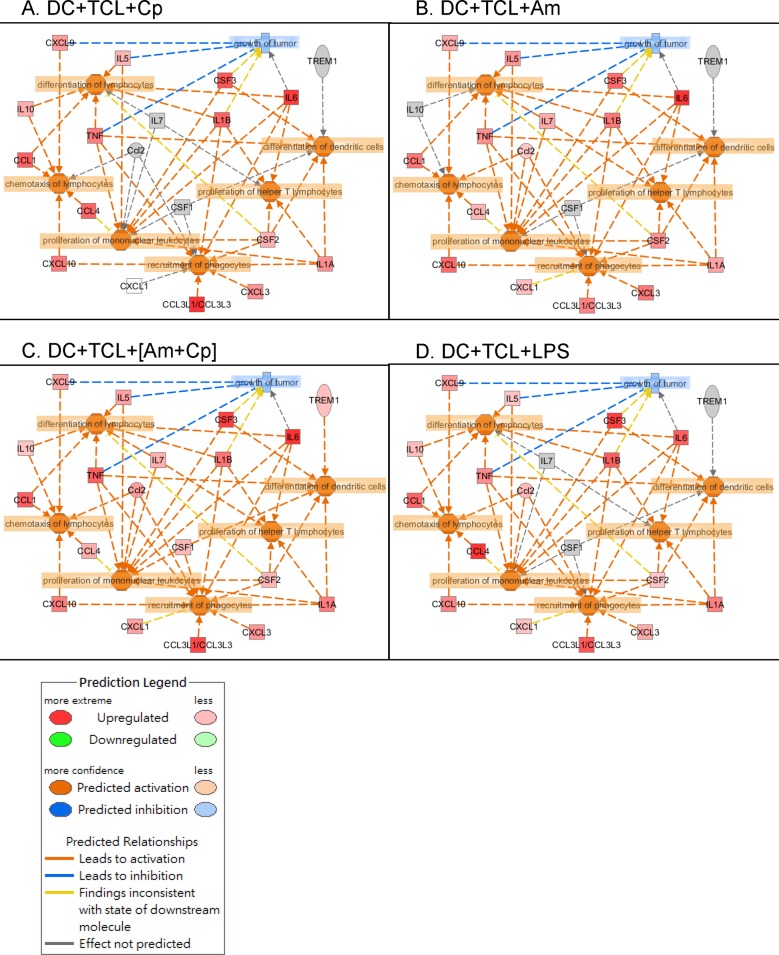
Functional pathway and immune cell-signaling analysis of TCL-loaded DCs which were treated with Cp-, Am-, [Am+Cp]- or LPS. The relationships between the biological functions and cytokines/chemokines of (A) DC+TCL+Cp, (B) DC+TCL+Am, (C) DC+TCL+[Am+Cp] and (D) DC+TCL+LPS were analyzed using Ingenuity Pathway Analysis (IPA) software. The cytokine/chemokine expression was determined by specific cytokine array (R&D Systems, Minneapolis, MN) and the expression levels that were altered more than two-fold in TCL groups were used in an IPA study. The signaling hubs (functions) include the differentiation of lymphocytes and dendritic cells, proliferation of helper T cells and monocytes, recruitment of phagocytes, chemotaxis of lymphocytes and inhibition of tumor growth.

Next, Ingenuity Pathway Analysis (IPA) software was used to analyze pathways, signaling and networks to reveal the possible functional significance of Cp, Am and [Am+Cp] as vaccine adjuvants in tested DCs. The differential expression of cytokines/chemokines in DCs with Cp-, Am-, [Am+Cp]-treatment and LPS-induction were identified by cytokine array analysis. Only the expression patterns altered more than two-fold were screened and selected for functional analysis. The IPA predictions suggested that DCs treated with Cp, Am, [Am+Cp] or LPS have drastically increased numbers of molecular markers for specific immune functions of TCL-loaded DCs. These include the differentiation of DCs, differentiation of lymphocytes, proliferation of help T lymphocytes, proliferation of mononuclear leukocytes, chemotaxis of lymphocytes and recruitment of phagocytes. These cellular physiological and immunological activities are ranked as the most significant functions in the networks related to the 19 molecules that are most highly upregulated in DCs with test phytochemicals (Fig 5). It is interesting to observe that the three test compounds (Cp, Am, [Am+Cp]), that are highly purified plant polysaccharides, exhibited a highly similar expression pattern for the 19 specific cytokines/chemokines, as that detected for the commonly used laboratory agent LPS. This similarity included high levels of induction of the pro-inflammatory cytokines IL-6, G-CSF, CCL3, CCL1 and IL-1β, which showed a 7- to 15-fold change in expression for [Am+Cp]- and LPS-treated DCs. A slightly lower, but still strong induction (4- to 7-fold change) of these pro-inflammatory immune modifiers was seen in the Cp- and Am-treated groups. Although, in general, the expression patterns were highly similar, specific differences were detected between the LPS- and [Am+Cp]-treated DCs. For example, expression of CCL4 (2.68X vs. 18.41X) and TREM-1 (2.17X vs. 1.33X) for the [Am+Cp] group and LPS group, respectively. CCL4 is known for its inhibition of proliferation of mononuclear leukocytes and TREM-1 for differentiation of dendritic cells [34,35]. The differential or distinguishable effect between these two test agents may be an important topic for future studies on DCs and other leukocytes. Other “minor” differences observed among the molecular markers for specific immune functions in the Cp, Am, [Am+Cp] groups may also be significant, but they need to be evaluated in future studies. Furthermore, three of the prognostic cytokines/chemokines, CXCL9, IL-5, and TNF (Fig 5), when expressed at an increased level in specific tissue or cellular environment, may confer a downregulatory effect on tumor growth [36–38].

### Monosaccharide residue composition of the Cp and Am polysaccharides

**Fig 6:**
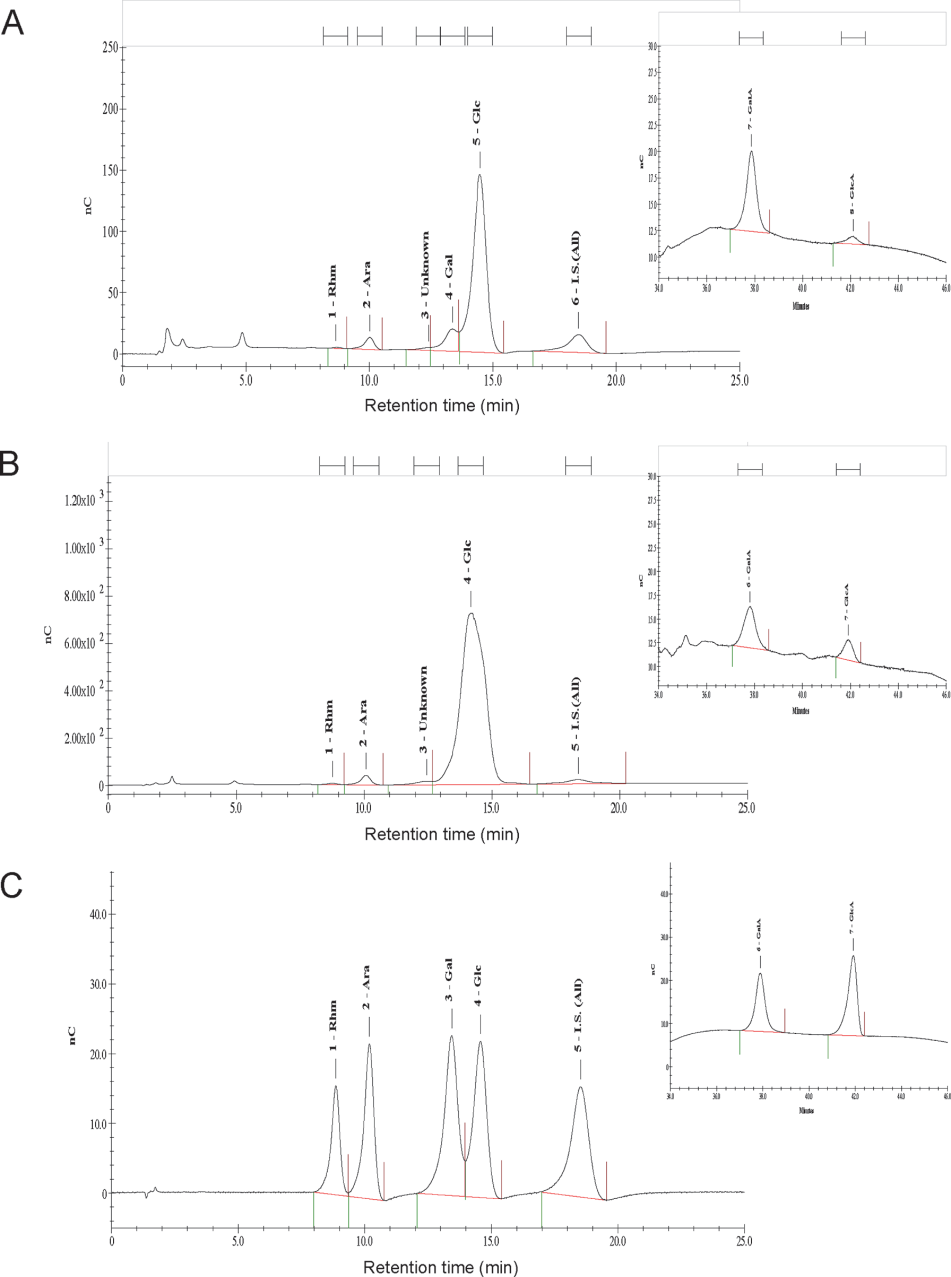
Chromatographic separation profile of the acid hydrolysate products of Cp or Am phytoextracts as analyzed by HPAEC-PAD. Monosaccharide composition of (A) Cp, (B) Am, and (C) mixture of seven monosaccharide standards; 1 = rhamnose (Rhm), 2 = arabinose (Ara), 3 = galactose (Gal), 4 = glucose (Glc), 5 = allose (All), 6 = galacturonic acid (GalA) and 7 = Glucuronic acid (GlcA).

The Cp and Am fractions were prepared, fractionated and highly purified as mixed polysaccharides with a molecular mass of 10 k to 150 k Daltons. The monosaccharide composition of Cp and Am phytoextracts, as ≥ 99% polysaccharides, were analyzed by high-performance anion-exchange chromatography (HPAEC). Typical chromatogram profiles of Cp and Am were obtained and are shown in Fig 6A and 6B, respectively. Specific peaks were identified by comparison of retention times with those of the reference monosaccharides (Fig 6C), determined by the injection of standard solutions and by standard addition (allose) of the analytes. The percentages of the carbohydrates and uronic acids present in Cp and Am samples are summarized in Table 2. Table 2 and Fig 6 show that the monosaccharide residues of Cp consist of glucose (70.23%), galactose (15.59%), galacturonic acid (8.26%), arabinose (4.69%), glucuronic acid (0.70%) and rhamnose (0.53%) (Table 2 and Fig 6A). In comparison, Am polysaccharides contain a high percentage (94.58%) of glucose residues and a little arabinose (3.04%), rhamnose (1.04%), galacturonic acid (0.99%) and glucuronic acid (0.38%) (Table 2 and Fig 6B). It is interesting to find that little (< 0.1%) or no mannose residue was detected in either of these plant polysaccharide preparations. Of note, Am did not contain any galactose, and yet Cp contains 15.59% galactose.

**Table 2:** Sugar composition of Cp and Am hydrolysate fractions.

**Phytoextract**	**Rhamnose**	**Arabinose**	**Galactose**	**Glucose**	**Galacturonic acid**	**Glucuronic acid**
**Cp**	0.53%	4.69%	15.59%	70.23%	8.26%	0.70%
**Am**	1.04%	3.04%	0%	94.58%	0.99%	0.38%

## Discussion

Traditional Chinese medicines have been used for the treatment of many diseases for more than 2,000 years, and increasingly evidence shows that co-administration of specific herbal medicines with conventional or standard western medicine therapies may reduce the side effects or/and improve the health of some patients with cancer or infectious diseases [39,40]. Recently, polysaccharides isolated from botanical sources, such as ginseng, mushrooms and yam, have received considerable attention due to their effect on measurable therapeutic properties and low toxicity, in both experimental animal and human systems [16,41,42]. Specific glucans have been reported to act as immune stimulating agents and effective T-cell immune adjuvants [42,43]. Some plant pectins confer anti-colon tumor, anti-allergy and anti-inflammation activities [43]. Arabinogalactans were reported to possess complement fixation activity and induced chemotaxis of human macrophages, T cells and NK cells [44]. Because of findings such as these, various plant polysaccharides are being considered as candidates for the development of immunomodulatory agents and adjuvants for immunotherapeutic DC vaccines [16,39,40,42]. Specific polysaccharides can possess immunomodulatory activities for DC activation via C-type lectins and lectin-like receptors [45,46], which are known to play important roles in various functions of DCs, including antigen uptake, T-cell interaction and migration [45]. Dectin-1, DC-associated C-type lectin-1 (Dectin-1), and DC-specific intercellular adhesion molecule-3-grabbing non-integrin (DC-SIGN), were all originally thought to be a type of DC-specific receptor, and are consistent with the potential role of these receptors in immune surveillance [45,47].

Increasing evidence has revealed that removing the primary tumor(s) by surgery may increase the risk of tumor metastasis [48–50]. Surgical trauma and the accompanying wound-healing processes may induce local and systemic changes in body physiology and immunity, including the disruption of structural integrity of tumor tissues and blood vessels, and the induction or increase in expression of inflammatory mediators and angiogenesis factors. These changes can lead to immunosuppression or/and enhanced adhesion or growth of tumor cells, which can, in turn, increase the chance of exfoliated tumor cells in developing local recurrences and distant metastases [50]. One of the strategies to prevent surgery-induced cancer metastasis is to heighten immune cell surveillance with cancer vaccines. Therefore, in this study, we evaluated the approach of using plant polysaccharides derived from Radix of Astragalus and Radix of Codonopsis as candidate adjuvants to increase the efficiency of a dendritic cell-based cancer vaccine, aiming to enhance the immune surveillance activity against metastatic tumor cells.

CD8^+^ T cells are known to play an important role in adaptive immune response to various cancers [51]. Costimulatory signals present on DCs are required for naive T cells to respond to specific antigenic stimulation. One key signal is delivered through the T cell receptor (TCR) by engagement with the peptide/MHC complexes, and a second signal is delivered by an interaction between the costimulatory molecules (such as CD28) and their ligands (e.g., CD80 and CD86) [52–54]. In this study, our results show that the candidate adjuvants (Am, Cp, and [Am+Cp]) can stimulate DCs to express increased levels of CD80 and CD86 molecules. Such enhanced expression of these molecules on DCs is known to promote the activation and proliferation of CD8^+^ T cells *in vitro*, as we have also shown here in Fig 1D. These activities are apparently associated with the increased anti-tumor efficacy of the tested DC-based vaccine in our *in vivo* study (Fig 2B and 2C). Increasingly, it has been reported that specific vaccines that can induce tumor-specific CD8^+^ T cell responses can provide a powerful approach for therapy against cancers [55,56]. These vaccines that introduce a specific tumor-associated antigen (TAA) to CD8^+^ T cells are often found to elicit a potent tumor-specific CD8^+^ T cell response against specific cancers. Furthermore, effective infiltration of CD8^+^ T cells into targeted tumors is known to suppress tumor cell growth [57]. Accumulation of infiltrating tumor-specific CD8^+^ T cells at the tumor site has been shown to increase antitumor efficacy [58]. In this study, our results (Fig 2F) indicate that the candidate adjuvants can augment the accumulation of CD8^+^ T cells in tumors established in the lung, and this may be associated with the increased antitumor/anti-metastasis activity of test DC-based vaccine *in vivo*. On the other hand, CD4^+^ T cells are known to cooperate with CD8^+^ T cells in specific immune responses. Stimulation of CD40 on APCs is through CD40L expression on helper CD4^+^ T cells which can activate and “license” the APCs to prime CD8^+^ T cell responses [59,60], and lead to enhanced tumor protection [61,62]. The result of Fig 3B[a] shows that CD40 was highly expressed in DCs treated with candidate adjuvants, and the proliferation of CD4^+^ T cells (Fig 1E) was significantly induced under specific adjuvant treatment. Taken together, we suggest that our candidate adjuvants, Am, Cp and [Am+Cp] can effectively stimulate DCs to express maturation markers for subsequent priming of both CD4^+^ and CD8^+^ T cell responses.

In this study, we demonstrated that Cp and Am contain mainly glucose-enriched polysaccharides, with few or no mannose residues (Table 2 and Fig 6). This finding is very different from that of the DsII polysaccharide sample, which we previously investigated [16] that contained a very high content (~53%) of mannan. We, therefore, suggest that specific glucans are presented in high abundance as the major compounds of polysaccharides Cp and Am. In terms of biological activities, a recent report indicated that specific glucans are capable of inducing the maturation of DCs by increasing the expression of maturation molecules in DCs, and these DCs can be stimulated by test glucans to activate T cell-mediated immunity [63]. Subsequently processes activated in this immunity can lead to activation of anti-tumor effector cells. These phytocompounds can, therefore, be effective for induction of anti-tumor immunity [43,63]. We would therefore like to suggest that the anti-tumor effect of the [Am+Cp]-stimulated, DC-based cancer vaccine may result from the bioactivity of glucans extracted from *Astragalus membranaceus* and *Codonopsis pilosulae* plants.

An *ex vivo* protocol now regarded as the “gold standard” for DC maturation, employs the use of a monocyte conditioned medium, based on promoting cell maturation with TNF-α, IL-1β, IL-6, and PGE2 [64]. This standard protocol and the DCs generated by this method have been used in a number of clinical studies [65,66]. By using specific phytocompounds from two medicinal plants in this study, we created a new *ex vivo* DC maturation protocol. In a very straightforward and simple way, polysaccharide mixtures of Am and Cp were shown to be effective inducers of a DC-based cancer vaccine against mammary carcinoma in test mice. Our data (Table 1 and Fig 5) show the secreted levels of IL-6, TNF-α, and IL-1β in treated DCs and their conditioned culture media. We suggest that such a drastic increase in IL-6, TNF-α and IL-1β expression in the culture medium provides a highly permissive cellular environment for effective maturation of [Am+Cp]-treated DCs circumventing the need for direct addition or supplement of these expensive pro-inflammatory cytokines into the DC medium. These results thus suggest an alternative choice of DC maturation agents, i.e., non-toxic plant-derived polysaccharides, to replace expensive/unstable recombinant cytokines or highly cytotoxic LPS. We suggest that [Am+Cp] is highly suitable for further development as a clinically useful adjuvant agent for DC-based vaccines, especially for health care and medical treatment needs in the undeveloped or developing world.

Increasing evidence shows that various cytokines produced by DCs can serve as key mediators for specific innate and adaptive immunities. For example, CCL1/CCR8 interaction can mediate the migration of DCs to the lymph nodes [67]. The DC-derived C-C chemokines, e.g., CCL3/CCL4, can act as chemotactic factors for preferentially attracting various inflammatory immune cells, including monocytes/macrophages, DCs, activated Th1 and Th2 cells, as well as NK cells and neutrophils to the sites of inflammation, and initiating an immune response [68–70]. In addition, mature DC-derived CXCL10 is known to play a pivotal role in retaining Th1 cells within the draining lymph nodes and in helping optimize the Th1-mediated immune responses [71]. In Table 1 and Fig 5, we revealed that [Am+Cp] can stimulate DCs to produce a number of the above-mentioned specific cytokines and chemokines, including CCL1/CCL3/CXCL10, which can effectively modulate the specific cellular functions of DCs. We may, therefore, conclude that [Am+Cp] not only enhances DC maturation activity, but also stimulates or induces the expression and secretion of a spectrum of DC-derived immumodulatory cytokines/chemokines.

Although the cellular/tissue microenvironment of cytokines/chemokines (e.g., drastic elevation or induction) is very important for the maturation and functions of DCs, excessive expression of specific cytokines/chemokines is known to generate a “cytokine storm” which can cause high fever, swelling, redness, extreme fatigue and nausea, an even death in normal or diseased humans [72]. As compared to [Am+Cp] treatment, our cytokine array data (Table 1 and Fig 5) shows that LPS treatment stimulated DCs to produce extremely high levels of several pro-inflammatory cytokines/chemokines such as G-CSF, CCL3, CCL4 and IL-1β. This cytokine profile difference may warrant future investigation for correlation with “cytokine storm”. It is interesting to observe that the inductions of TNF-α and IL-6 for both LPS and [Am+Cp] treatments appear to be quite similar. Therefore, we believe that [Am+Cp] may be more useful as a clinical activating agent than LPS or recombinant pro-inflammatory cytokines for dendritic cell-based cancer vaccine against metastatic mammary cancers.

## Supporting Information

S1 ARRIVE checklist ARRIVE Checklist. (PDF)

S1 Fig The four parameters, forward scatter (FSC), side scatter (SSC), log FITC (MHC II) and log PE (CD11c), for gating cell flow cytometry assays were simultaneously collected. (A) The purity of DC populations was determined by flow cytometry analysis. Our culture procedure/conditions routinely resulted in ≥ 85% MHC-II^+^ and CD11c^+^ DCs of the total cultured cell population. (B) The gating scope (P9) was defined to exclude debris and aggregated cells on a FSC/SSC histogram; 10,000 cells were counted in P9 and data were recorded.
(TIF)

S2 Fig Images of MTT formazan crystals present on and in test DCs. BMDCs (2 × 10^5^) were cultured in “complete culture medium” only (A), or with complete medium containing 200 μg/ml Cp (B), Am (C) or [Am+Cp] (D) for 24 h, followed by 4 additional hours of MTT reduction (0.5 mg/ml). Test cells then were examined and photographed with a Nikon light microscope. The red arrows indicate cells with needle-like MTT formazan crystals.
(TIF)

S3 Fig (A) Cytokine array panel coordinates (B) Information on all 40 cytokines/chemokines tested in the cytokine array. (TIF)

S4 Fig Effect of tumor cell lysate at concentrations between 50 and 1000 μg/ml on viability of mouse splenocyte cells. (TIF)

S5 Fig Flow cytometry analysis of expression of MHC class II on DCs subjected to different treatments. The untreated DCs were harvested on day 7 (A) and day 8 (B)[a] post cell cultivation. Some replicate sets of day 7 DC cultures were treated with TCL for only 24 h (B)[b] or treated with TCL for 2 h, and then activated with 200 μg/ml of Cp (B)[c], Am (B)[d], [Am+Cp] (B)[e] or 1 μg/ml of LPS (B)[f] for another 22 hours. Subsequently, MHC class II expression on DCs from different treatment sets were analyzed by flow cytometry.
(TIF)

S1 File Guidelines for determining endpoints and humane termination of animals. (PDF)

S2 File Approval letter. This is to certify that the animal protocol by the following applicant has been evaluated and approved by the Institutional Animal Care and Use Committee of Academia Sinica (AS IACUC).
(PDF)

S1 Table Stimulation (in fold change) of all 40 cytokines and chemokines in DCs-treated with Cp, Am, [Am+Cp] or LPS groups compared to control group. (PDF)
