# Supplementary figures and images for: Specific Medicinal Plant Polysaccharides Effectively Enhance the Potency of a DC-Based Vaccine against Mouse Mammary Tumor Metastasis
Source: PLoS One. 2015 Mar 31;10(3):e0122374. doi: 10.1371/journal.pone.0122374 (PMC4380423; doi:10.1371/journal.pone.0122374)

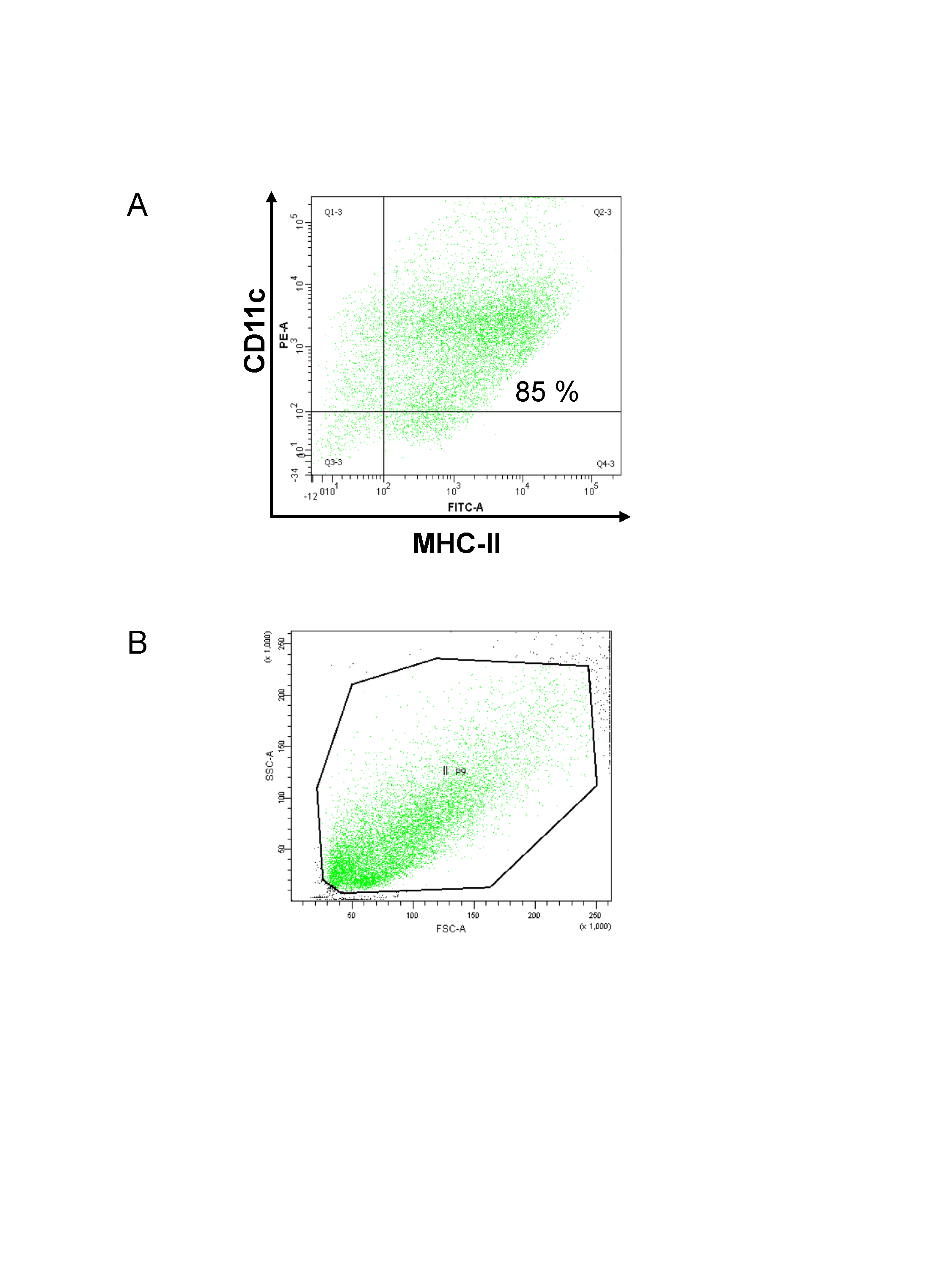

Supplement: S1 Fig — (A) The purity of DC populations was determined by flow cytometry analysis. Our culture procedure/conditions routinely resulted in ≥ 85% MHC-II+ and CD11c+ DCs of the total cultured cell population. (B) The gating scope (P9) was defined to exclude debris and aggregated cells on a FSC/SSC histogram; 10,000 cells were counted in P9 and data were recorded. (TIF) [file pone.0122374.s002.tif]

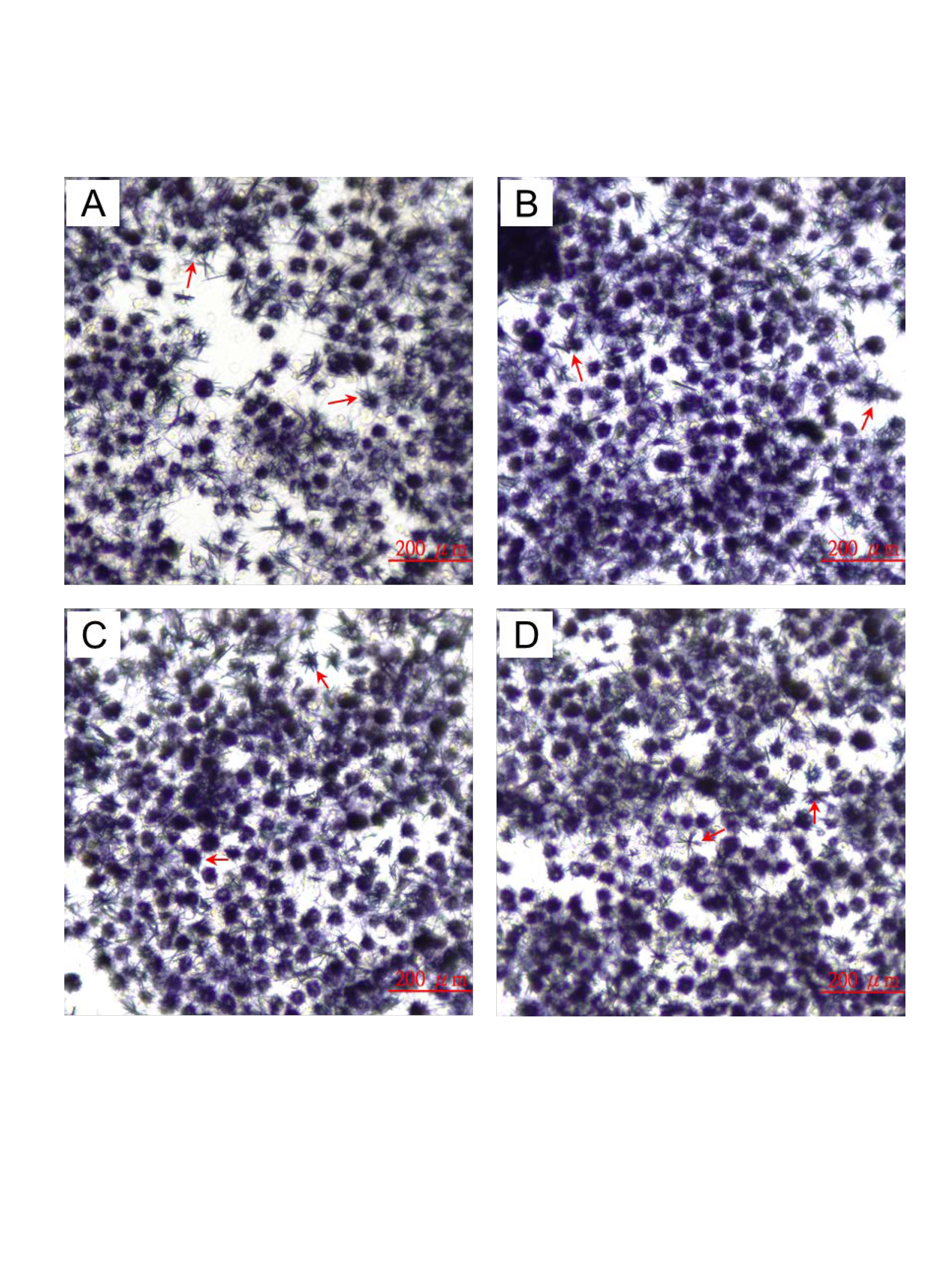

Supplement: S2 Fig — BMDCs (2 × 10^5^) were cultured in “complete culture medium” only (A), or with complete medium containing 200 μg/ml Cp (B), Am (C) or [Am+Cp] (D) for 24 h, followed by 4 additional hours of MTT reduction (0.5 mg/ml). Test cells then were examined and photographed with a Nikon light microscope. The red arrows indicate cells with needle-like MTT formazan crystals. (TIF) [file pone.0122374.s003.tif]

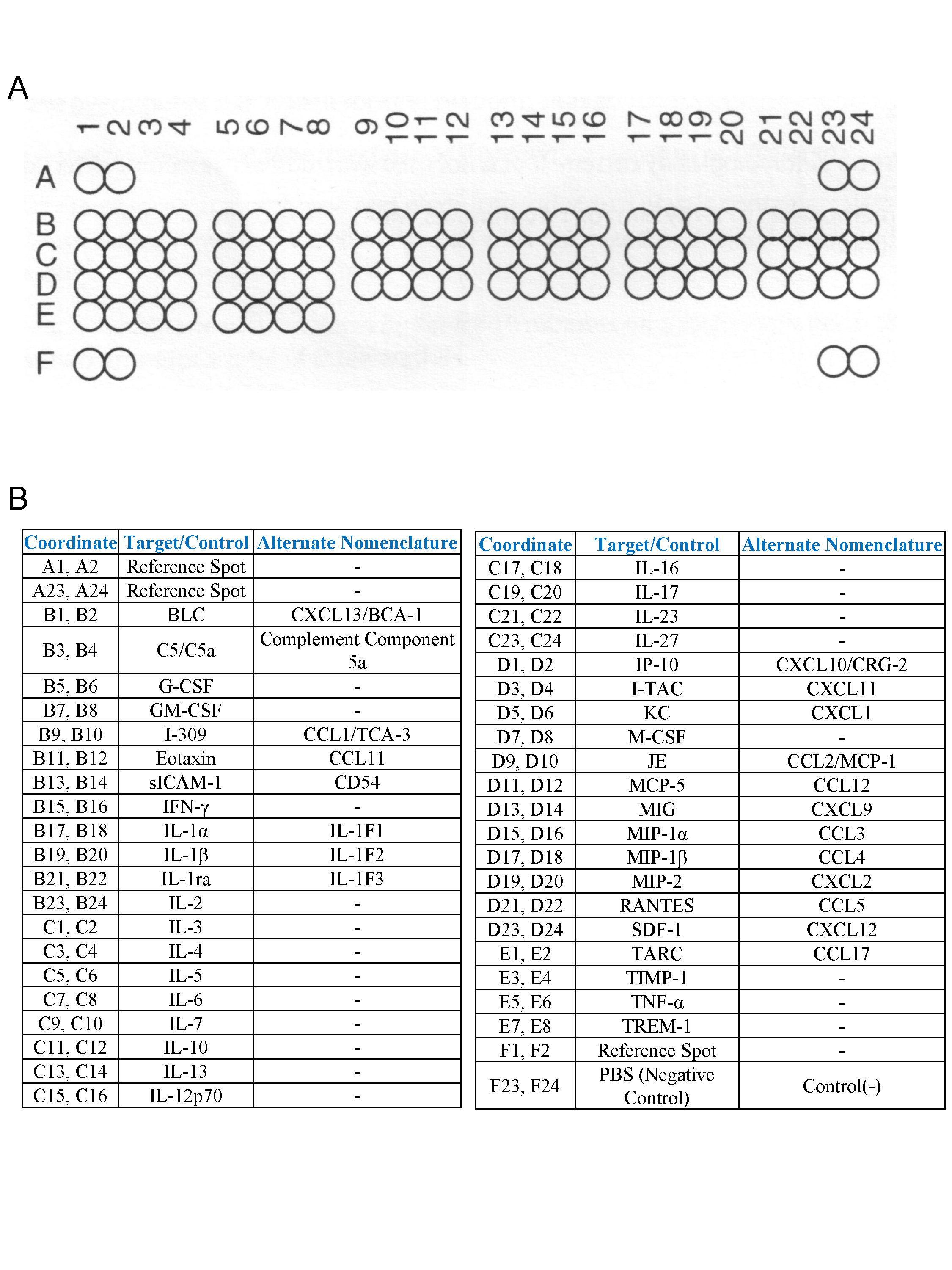

Supplement: S3 Fig — (TIF) [file pone.0122374.s004.tif]

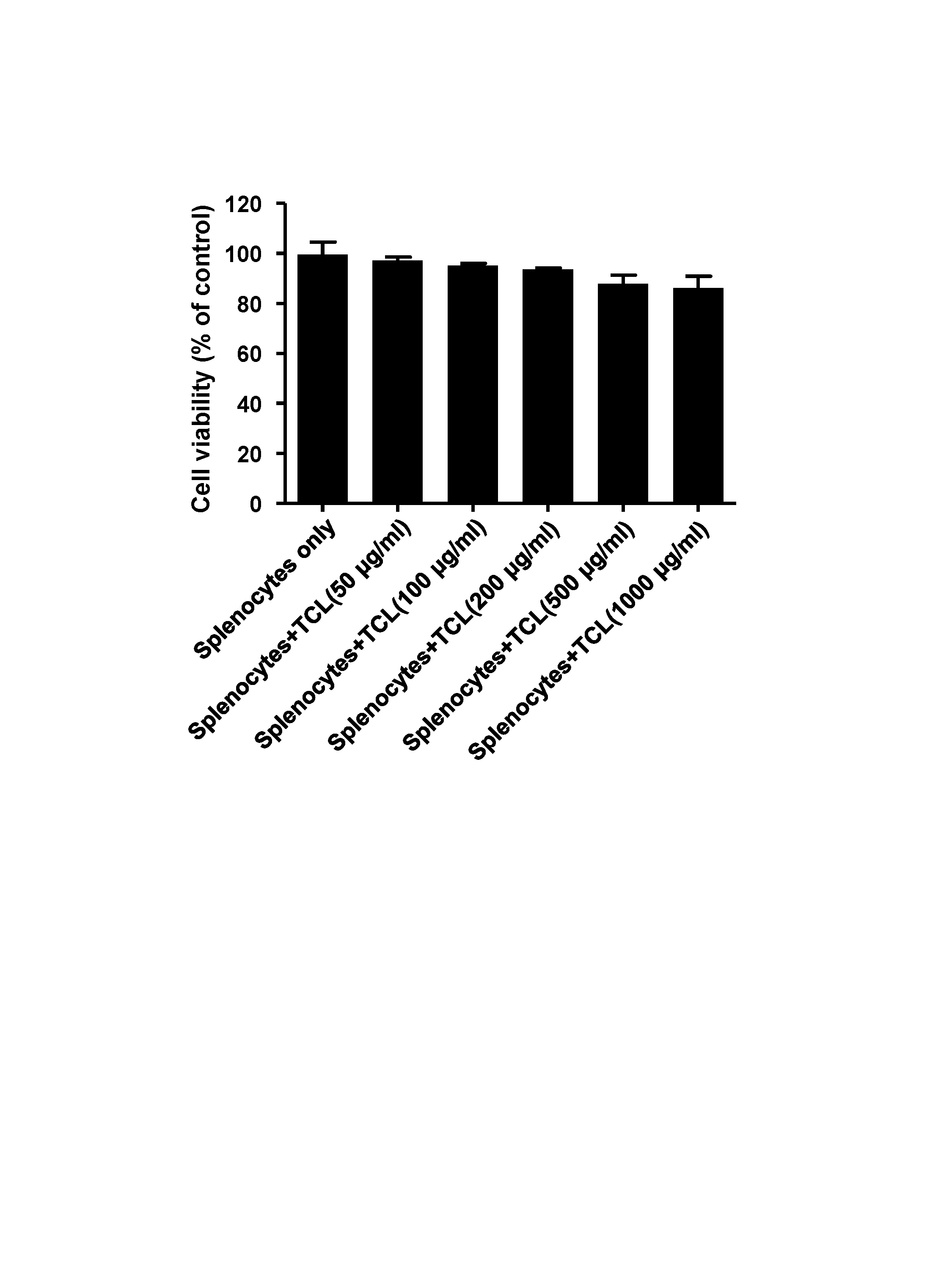

Supplement: S4 Fig — (TIF) [file pone.0122374.s005.tif]

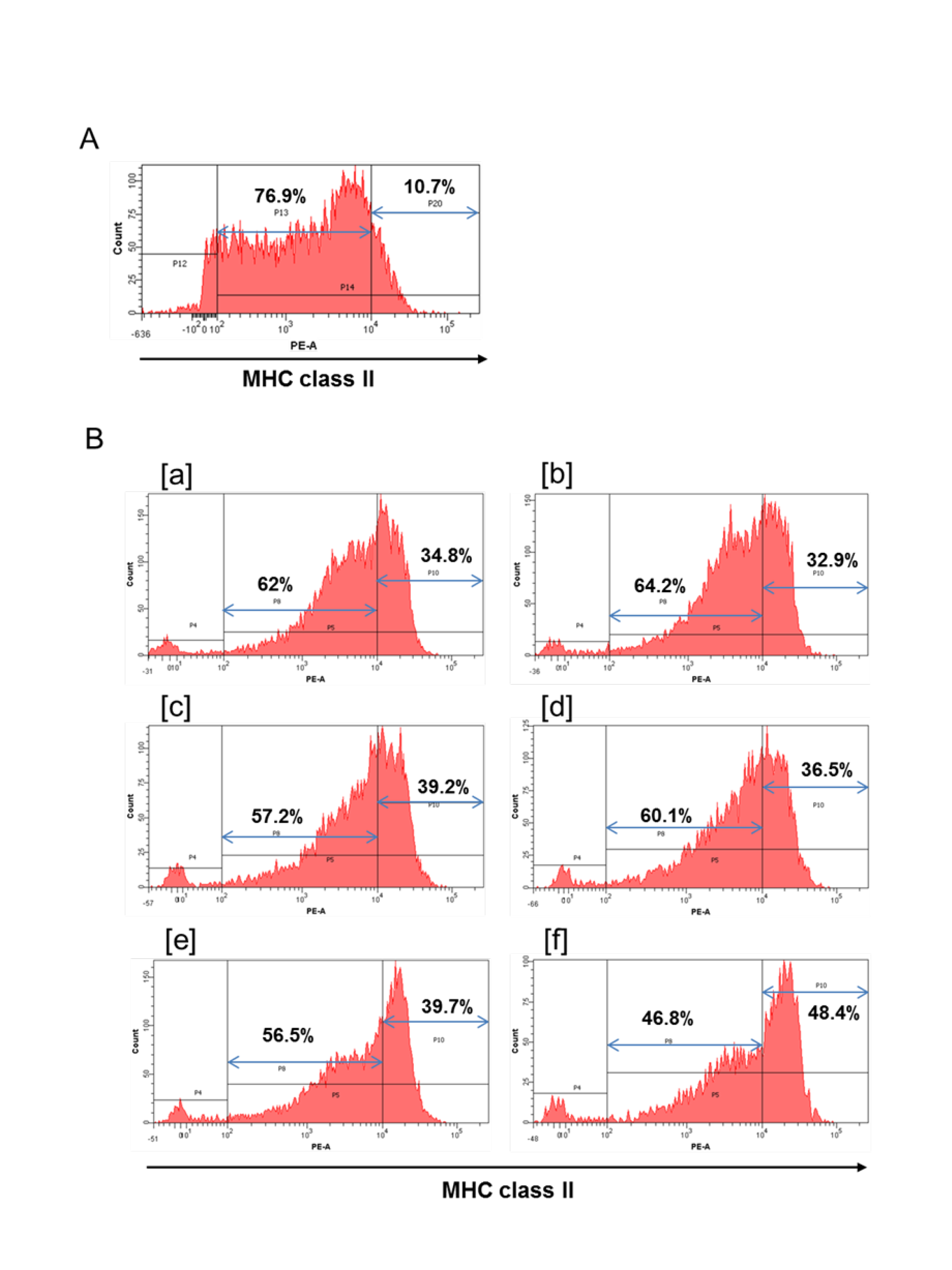

Supplement: S5 Fig — The untreated DCs were harvested on day 7 (A) and day 8 (B)[a] post cell cultivation. Some replicate sets of day 7 DC cultures were treated with TCL for only 24 h (B)[b] or treated with TCL for 2 h, and then activated with 200 μg/ml of Cp (B)[c], Am (B)[d], [Am+Cp] (B)[e] or 1 μg/ml of LPS (B)[f] for another 22 hours. Subsequently, MHC class II expression on DCs from different treatment sets were analyzed by flow cytometry. (TIF) [file pone.0122374.s006.tif]
