# Supplementary material for: Specific Medicinal Plant Polysaccharides Effectively Enhance the Potency of a DC-Based Vaccine against Mouse Mammary Tumor Metastasis
Source: PLoS One. 2015 Mar 31;10(3):e0122374. doi: 10.1371/journal.pone.0122374 (PMC4380423; doi:10.1371/journal.pone.0122374)
Supplement: S1 File — (PDF) [file pone.0122374.s007.pdf]

# GUIDELINES FOR DETERMINING ENDPOINTS AND HUMANE TERMINATION OF ANIMALS

Animals exhibiting clinical signs of pain or distress must be euthanized unless other measures will be taken to alleviate these symptoms (e.g. supportive care, appropriate treatment for the specific condition) or scientific justification for no alleviation or euthanasia has been provided by the investigator and approved by the IACUC.

Judgment regarding when an animal is to be euthanized should be based on the seriousness of the animal's condition (e.g. severity/number of clinical signs, ability of the animal to recover from the condition).

Where uncertainty about the need for euthanasia or treatment exists, the LAS veterinary staff must be consulted. The following criteria for euthanasia may be used collectively or individually, as appropriate for the study and the condition of the animal:

1. Decreased food or water consumption (e.g. the animal fails to eat and/or drink for more than 24 hours)
2. Inability to ambulate to reach food or water.
3. Abnormal urine (e.g. markedly discolored, hematuria)/decreased or increased urine output.
4. Chronic and/or severe diarrhea or constipation.
5. Weight loss (more significant if rapid; if gradual [e.g. over 7 days] categorized as significant if >20%. Body condition scoring is also useful in conjunction with weight loss.
6. Dehydration (e.g. skin tenting, sunken eyes).
7. Hypothermia .
8. Marked change in behavior/depression (e.g. lethargy, non-responsiveness, abnormal vocalization, aggression).
9. Rough hair coat/hunched posture in conjuction with other clinical signs and especially if debilitating or prolonged (3 days).
10. Distended abdomen in conjunction with other clinical signs and in association in conditions that lead to debilitation (e.g. neoplasia, liver failure).
11. Progressive respiratory distress (e.g. difficulty breathing, coughing, rales, wheezing).
12. Abnormal mucous membrane color (e.g. pale/blue-tinged mucous membranes).
13. Abnormal discharge (e.g. nasal discharge, draining lesion).
14. Distinct icterus (e.g. yellowish coloring of the skin or tissues) .
15. Severe and/or uncontrollable bleeding.
16. Central nervous system signs such as head tilt, tremors, spasticity, seizures, circling or paralysis or paresis, especially if associated with anorexia .
17. Cardiovascular disease with related clinical signs (e.g. coughing, respiratory distress, cyanosis, limb edema).
18. Persistent self-induced trauma.
19. Abnormalities of the skin and/or musculoskeletal system (e.g. swelling, redness, discoloration, evidence of pain on palpation, ulceration, abnormally warm or cold to the touch).
20. Porphyrin (red-tinged) staining around the eyes or nose in rodents (in conjunction with other clinical signs).
21. Rapid growth of a mass or clinical signs of neoplasia.
22. Lesions interfering with eating or drinking .
23. Hematologic or biochemical parameters that indicate organ failure incompatible with life.

24. Excessive tumor burden (tumors which exceed 15% of the animal's normal body weight or interferes with the animal's ability to eat, drink, or move normally).
25. Tumors that exhibit necrosis/ulceration.
26. Evidence of infection that is not readily treatable (e.g. evidence of pus, inflammation, swelling involving any area of the animal's body) or requiring necropsy for diagnosis.
27. Unconsciousness with no response to external stimuli such as handling or the toe-pinch withdrawal test.
28. Fluid accumulation in body cavities/subcutaneous tissue .
29. Untreatable rectal prolapse (the rectum falls from its normal position and protrudes from the anus).
30. Excessive licking, scratching, or signs of self-mutilation.

Other clinical signs judged by experienced technical staff to be indicative of morbidity or being in a moribund condition .

Definitions:

Morbidity: The condition of being diseased or unhealthy

Moribund: in a dying state.
