# Supplementary material for: Specific Medicinal Plant Polysaccharides Effectively Enhance the Potency of a DC-Based Vaccine against Mouse Mammary Tumor Metastasis
Source: PLoS One. 2015 Mar 31;10(3):e0122374. doi: 10.1371/journal.pone.0122374 (PMC4380423; doi:10.1371/journal.pone.0122374)
Supplement: S2 File — This is to certify that the animal protocol by the following applicant has been evaluated and approved by the Institutional Animal Care and Use Committee of Academia Sinica (AS IACUC). (PDF) [file pone.0122374.s008.pdf]

eProtocol Admin.
Dr. Fu-Hwa Liu.
Tel: (02) 2789-8700
iacuc@gate.sinica.edu.tw

Dear 楊, 寧蓀,

茲證明此動物實驗計畫書(如下列)業經中央研究院動物實驗管理委員會審核通過。

This is to certify that the animal protocol by the following applicant has been evaluated and approved by the Institutional Animal Care and Use Committee of Academia Sinica(AS IACUC).

Protocol ID: 12-01-304
Principal Investigator: 楊, 寧蓀
Department: 農生中心(ABRC)
Protocol Title: Enhancement of the adjuvant effect of selected phytochemicals on dendritic cell (DC)-based cancer vaccines in preventing cancer metastasis into lung
Funding :國科會(NSC),Start Date : 05/01/2012, End Date :04/30/2015
Approval Date: 04/19/2012
Period of Total Care and Use Period：04/19/2012 to 04/18/2013
Animal Species/Strains:Mouse / BALB/c (Inbred) , Mouse / Athymic Nude (Immunodeficient)
Number to be used per year: 180~ 180

Sincerely,
中央研究院動物實驗管理委員會召集人 孔祥智

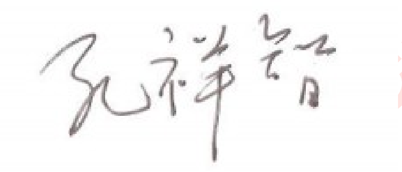

John T. Kung Ph.D., Chair, AS IACUC

e-protocol
