# Supplementary material for: Specific Medicinal Plant Polysaccharides Effectively Enhance the Potency of a DC-Based Vaccine against Mouse Mammary Tumor Metastasis
Source: PLoS One. 2015 Mar 31;10(3):e0122374. doi: 10.1371/journal.pone.0122374 (PMC4380423; doi:10.1371/journal.pone.0122374)
Supplement: S1 Table — (PDF) [file pone.0122374.s009.pdf]

S1 Table. Stimulation (in fold change*) of all 40 cytokines and chemokines in DCs-treated with Cp, Am, [Am+Cp] or LPS groups compared to control group**

	Cytokines/Chemokines	Alternate Nomenclature	Cp	Am	[Am+Cp]	LPS
1	IL-6	–	10.23	14.18	12.12	9.95
2	G-CSF	–	7.37	6.82	10.60	15.58
3	MIP-1α	CCL3	11.15	4.96	9.16	10.74
4	I-309	CCL1/TCA-3	6.61	5.93	7.11	9.03
5	TNF-α	–	4.62	4.32	6.99	5.16
6	IL-1β	IL-1F2	6.36	5.89	5.38	9.86
7	IL-1α	IL-1F1	3.75	3.27	4.95	6.08
8	IP-10	CXCL10/CRG-2	4.85	6.82	4.80	5.59
9	MIP-2	CXCL2	3.62	5.81	3.62	3.31
10	JE	CCL2M/CP-1	1.80	2.08	3.31	2.71
11	MIG	CXCL9	3.24	2.90	3.12	4.13
12	KC	CXCL1	1.72	2.37	3.12	2.38
13	IL-5	–	2.42	3.92	3.11	2.29
14	MIP-1β	CCL4	6.15	2.66	2.68	18.41
15	IL-7	–	1.85	3.05	2.64	1.89
16	GM-CSF	–	2.51	4.31	2.49	2.27
17	M-CSF	–	1.46	1.93	2.35	1.57
18	TREM-1	–	1.24	1.43	2.17	1.33
19	IL-10	–	2.63	1.96	2.02	2.59
20	I-TAC	CXCL11	1.76	2.96	1.90	1.93
21	C5/C5a	Complement Component 5a	1.23	1.71	1.69	1.31
22	IL-17	–	2.10	3.63	1.68	1.92
23	MCP-5	CCL12	1.25	1.89	1.67	1.38
24	IL-3	–	1.19	2.44	1.27	1.51
25	IL-4	–	0.89	1.33	1.17	0.97
26	sICAM-1	CD54	1.04	1.44	1.12	0.80
27	BLC	CXCL13/BCA1	0.98	1.70	1.09	0.97
28	TIMP-1	–	0.64	1.01	1.09	1.03
29	Eotaxin	CCL11	1.31	1.71	1.00	0.85
30	TARC	CCL17	0.82	1.39	0.97	0.91
31	IL-12p70	–	1.91	2.48	0.96	1.09
32	IL-27	–	1.13	1.49	0.96	0.80
33	SDF-1	CXCL12	1.03	1.39	0.95	0.47
34	IL-23	–	1.13	1.74	0.94	0.85
35	IFN-γ	–	1.14	1.66	0.92	0.92
36	IL-13	–	1.48	1.87	0.91	0.72
37	RANTES	CCL5	0.64	0.87	0.87	0.68
38	IL-16	–	0.79	1.02	0.81	0.73
39	IL-2	–	1.15	1.84	0.80	0.53
40	IL-1ra	IL-1F3	1.02	1.45	0.77	0.73

* Fold change = treatment group/control untreated group

** Control group: DC+TCL
